# Native valve, prosthetic valve, and cardiac device-related infective endocarditis: A review and update on current innovative diagnostic and therapeutic strategies

**DOI:** 10.3389/fcell.2022.995508

**Published:** 2022-10-03

**Authors:** Joop J. P. Kouijzer, Daniëlle J. Noordermeer, Wouter J. van Leeuwen, Nelianne J. Verkaik, Kirby R. Lattwein

**Affiliations:** ^1^ Thoraxcenter, Department of Biomedical Engineering, Erasmus MC University Medical Center, Rotterdam, Netherlands; ^2^ Department of Cardiothoracic Surgery, Erasmus MC University Medical Center, Rotterdam, Netherlands; ^3^ Department of Medical Microbiology and Infectious Diseases, Erasmus MC University Medical Center, Rotterdam, Netherlands

**Keywords:** biofilm, cardiac device, diagnosis, infection, infective endocarditis, native valve, prosthetic valve, treatment

## Abstract

Infective endocarditis (IE) is a life-threatening microbial infection of native and prosthetic heart valves, endocardial surface, and/or indwelling cardiac device. Prevalence of IE is increasing and mortality has not significantly improved despite technological advances. This review provides an updated overview using recent literature on the clinical presentation, diagnosis, imaging, causative pathogens, treatment, and outcomes in native valve, prosthetic valve, and cardiac device-related IE. In addition, the experimental approaches used in IE research to improve the understanding of disease mechanisms and the current diagnostic pipelines are discussed, as well as potential innovative diagnostic and therapeutic strategies. This will ultimately help towards deriving better diagnostic tools and treatments to improve IE patient outcomes.

## Introduction

This review provides an overview on native valve infective endocarditis (NVE), prosthetic valve (PVE) and cardiac device-related infective endocarditis (CDRIE) concerning epidemiology, pathogenesis, clinical presentation, diagnosis, causative microbes, and treatments with outcomes. Articles published within approximately the last 5 years (from 2016) were included for providing information pertaining to diagnosis, treatment, and outcomes. Literature published in the last 10 years (from 2011) was also included when more recent literature concerning symptoms, causative agents, and treatment options was unavailable. The latter part of the review presents the current and new investigative developments with respect to IE, as well as briefly discusses potential new avenues for diagnostic pipelines, therapeutic strategies, and research methods.

### Epidemiology and risk factors

**FIGURE 1:**
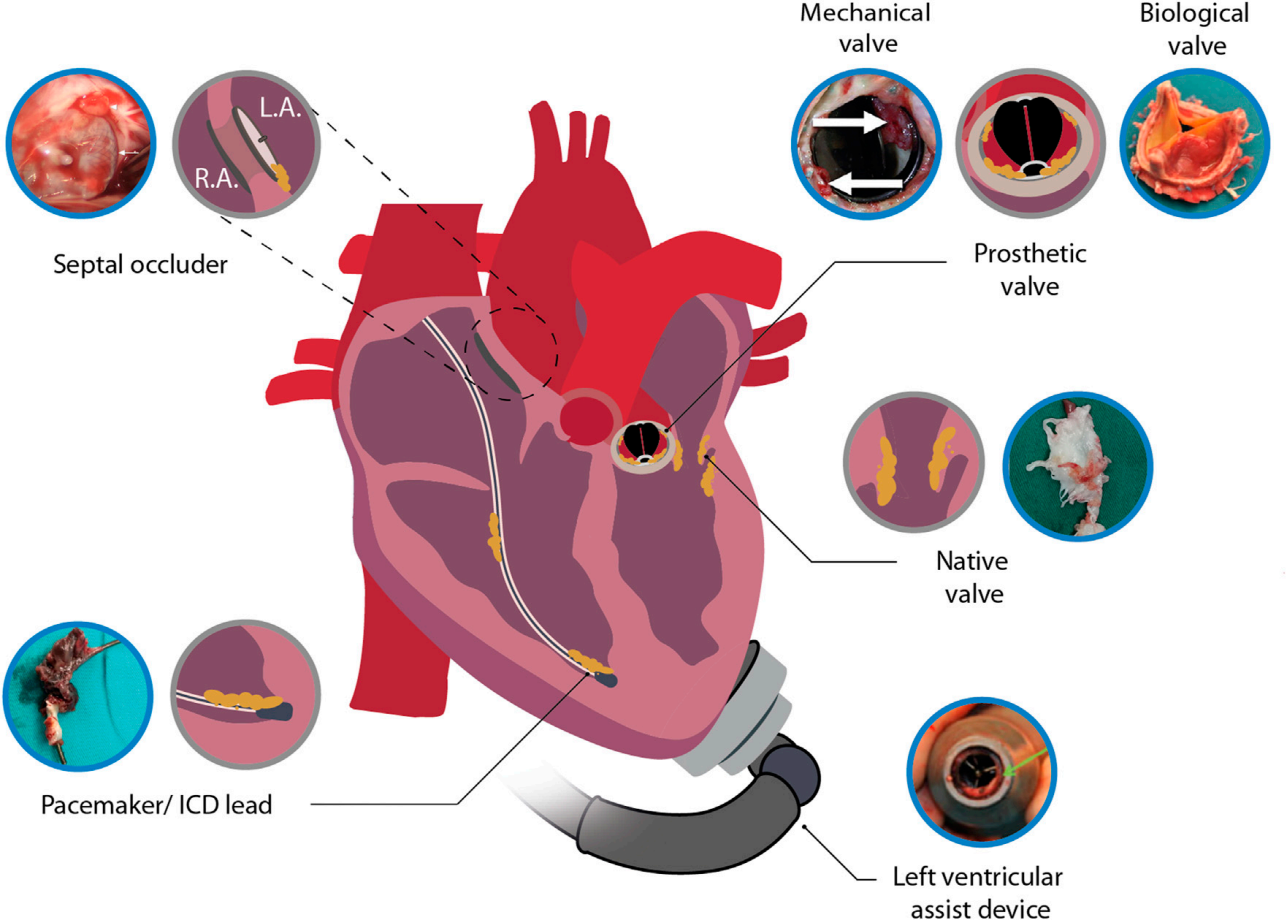
Illustration depicting infective endocarditis associated with native heart tissue and cardiac devices. Included images are examples of an infected septal occluder (Nguyen et al., 2016), mechanical heart valve (Pettersson et al., 2014), biological prosthetic heart valve (Jainandunsing et al., 2014), native heart valve (Li et al., 2022), LVAD (Akin et al., 2018) and pacemaker lead (Boljevic et al., 2019). Blue encircled images were adapted with permission from the original publishers and used as examples. L.A., left atrium; R.A., right atrium.

Infective endocarditis (IE) is a life-threatening microbial infection of the native and prosthetic valves, endocardial surface, or indwelling cardiac device (Figure 1). Despite advances in diagnosis and management, IE is associated with high mortality (6–50% in-hospital mortality and 19–82% 5-year mortality) (Abegaz et al., 2017; Mistiaen, 2018; Habib et al., 2019; Suzuki et al., 2019; Witten et al., 2019; El Kadi et al., 2020; Huuskonen et al., 2021) which has not significantly improved over the last decades. The prevalence of IE, currently at 5–14.3 per 100,000 per year among the adult population (Thornhill et al., 2020; Williams et al., 2021), is increasing (Keller et al., 2017; Ahtela et al., 2019; Shah A. S. V. et al., 2020) and in some countries has doubled over the past 10 years (van den Brink et al., 2017; Thornhill et al., 2020). This increase is largely attributed to the growing population with age-related degenerative valvular disease, chronic co-morbidities, and an increased need for invasive procedures and implanted cardiac devices (Forestier et al., 2016; Wu et al., 2019; Shah A. S. V. et al., 2020). Risk factors for developing IE include cardiac risks, such as degenerative valvular disease, congenital valvular abnormalities, rheumatic heart disease, atrial fibrillation, post cardiac transplant valvulopathy and/or indwelling cardiac devices, as well as non-cardiac risks, such as intravenous drug use, poor dentition, chronic liver disease, hemodialysis and/or advanced age (Bin Abdulhak et al., 2018; Yang and Frazee, 2018; Chambers and Bayer, 2020). Further risk factors specific for IE following aortic valve replacement (surgical or transcatheter) include younger age and male sex, as well as an elevated body mass index specific for surgical replacement and an elevated post-deployment gradient and self-expanding valves for transcatheter replacement (Cahill et al., 2022).

### Pathogenesis

Concerning IE pathogenesis, bacteria possess several adherence factors that facilitate direct microbial attachment to the surfaces within the cardiovascular system, whether that be tissue or device (Cahill and Prendergast, 2016). IE development on native tissue is understood to be initiated by valvular endothelium damage or inflammation to which pathogens adhere directly or indirectly by fibrin deposition or the activated endothelial layer (Holland et al., 2016; Liesenborghs et al., 2019; Liesenborghs et al., 2020; Lerche et al., 2021). Activated endothelial cells can initiate the deposition of von Willebrand factor, which subsequently recruits platelets leading to the formation of fibrin. The presence of von Willebrand factor multimers and fibrin are believed to play an important role in the initial adherence of bacteria (Claes et al., 2014; Liesenborghs et al., 2019). For infection development on indwelling leads and devices, blood protein deposition and disturbed blood flow provide niches for bacterial adherence or contamination can occur with a patient’s own skin flora at the time of implantation (Blomström-Lundqvist et al., 2020). Once adhered, bacteria encase themselves in a protective extracellular matrix, consisting of numerous bacterial and host components, such as fibrin, platelets, host and bacterial proteins, polysaccharides, and extracellular DNA. The combination of both the bacteria and extracellular matrix is called a biofilm, also commonly known as a vegetation in the clinical setting when it can be seen macroscopically. Biofilm confers several benefits for bacteria which impact treatment success, such as tight endothelial adherence and protection against the immune system and antimicrobial treatments (Werdan et al., 2014; Holland et al., 2016).

## Clinical presentation

The clinical presentation of IE is diverse, ranging from severe infection to indolent with nonspecific symptoms (Cahill et al., 2017; Yang and Frazee, 2018). IE presentation can be divided into subacute and acute (Vincent and Otto, 2018; Ibrahim and Siddique, 2021). Subacute infection has a gradual development of disease over the course of weeks to months and is associated with nonspecific symptoms, such as fever, chills, sweats, dyspnea, and back pain. Acute infection has a more sudden onset that can become life-threatening within days and is associated with severe clinical signs, such as sepsis, stroke, and pulmonary and systemic embolization. Heart failure, with new-onset more common than worsening pre-existing, can also occur in acute IE infections due to severe valvular obstruction or insufficiency (Pericas et al., 2021). Subacute endocarditis is often marked by delayed diagnosis, up to 40 days with a median delay of 13 days (Nishiguchi et al., 2020). This is largely attributed to the nonspecific symptoms that prove difficult to pinpoint the disease and arrive at a definitive diagnosis of IE (Nishiguchi et al., 2020). The most reported presenting symptoms are summarized in Table 1. For NVE, PVE, and CDRIE, fever and cardiac murmur were the most common. Further symptoms include cough, embolization, heart failure, sepsis, stroke, and immunologic phenomena, such as Osler’s nodes, Janeway lesions, and Roth’s spots, are rarely found. Subacute and acute presentation can be linked to the most relevant bacteria responsible for this type of presentation. The division of subacute infection is more often linked to streptococci and acute infection to staphylococci, with *S. aureus* most common (Talha et al., 2020). *S. aureus* has become the most common infecting bacteria causing IE and is associated with higher mortality, twice as long hospitalization times, and neurological complications (Tong et al., 2015; Das et al., 2022).

**TABLE 1:** Presenting symptoms in IE.

Symptoms	Abscess	Chills	Cough	Dyspnea	Embolization	Fever	Heart failure	Inflammation in area	Janeway lesions	Malaise	Murmur	Osler nodes	Pulmonary infection	Roth spots	Sepsis	Stroke
NVE																
Babes et al. (2021)																
De Camargo et al. (2019)																
Habib et al. (2019)																
Servy et al., 2014																
CDRIE																
Granados et al. (2016)																
Habib et al. (2019)																
Jędrzejczyk-Patej et al. (2021)																
Plonska-Gosciniak et al., 2019																
Saez et al., 2019																
PVE																
De Camargo et al. (2019)																
Habib et al. (2019)																
Selton-Suty et al., 2012																
Undefined IE																
Ortiz-Bautista et al. (2017)																
Zaqout et al., 2020																

Green = common (≥50%); orange = sometimes (10–50%); red = uncommon (≤10%).

CDRIE, cardiac device-related infective endocarditis; IE, infective endocarditis; NVE, native valve endocarditis; PVE, prosthetic valve endocarditis.

## Diagnosis

For diagnostic classification, the modified Duke criteria are used, with diagnosis categories of definite (two major or one major and three minor), possible (one major and one minor or three minor) or rejected IE (Gomes et al., 2017; Horgan et al., 2020a). The major criteria include positive blood culture, positive echocardiography, and valvular regurgitation (Cahill and Prendergast, 2016; Vincent and Otto, 2018; Blomström-Lundqvist et al., 2020; Hubers et al., 2020). Additional criteria have been recommended in the diagnosis of CDRIE patients, which can be found in the European Heart Rhythm Association consensus statement (Blomstrom-Lundqvist et al., 2020). The sensitivity of the modified Duke criteria to provide a definite IE diagnosis is 80% in native valves, and 70% in cardiac devices (Cahill and Prendergast, 2016; Gomes et al., 2017). Concerning only heart valve associated IE, both native and prosthetic, the sensitivity has been reported to be 72% and specificity at 74% (Shrestha et al., 2017).

### Microbiological diagnosis

Positive blood cultures remain crucial in the diagnosis of IE by demonstrating the presence of bacteria (Rajani and Klein, 2020). At least two positive blood cultures, from microorganisms typically known to cause IE, are required within at least 12 h between the first and last sample, or a set of three separate cultures obtained with at least 1 h between the first and last sample (Cahill and Prendergast, 2016; Liesman et al., 2017; Vincent and Otto, 2018; Blomström-Lundqvist et al., 2020; Hubers et al., 2020). In the case of no bacterial growth in combination with a high suspicion for IE, blood culture incubation should be prolonged and serological testing should be performed (Rajani and Klein, 2020). Tissue cultures are also used to determine the causative microorganism in patients with IE. Although blood and tissue cultures are considered the gold standard for pathogen identification, false negative results can occur (ranging from 2.5 to 31% (Brouqui and Raoult, 2001)). False negative test results are attributed to the use of antibiotic therapy prior to blood sample collection, bacteria that are difficult to culture, fastidious or grow intracellular (Houpikian and Raoult, 2005). Further, results of blood cultures can be inconclusive due to contamination by commensal bacteria, a complex polymicrobial biofilm (Oberbach et al., 2017), or insufficient amounts of bacteria to culture. Negative blood cultures are found in approximately 24% of all IE cases, which is similar for NVE (25%), CDRIE (26%), and PVE (23%) (Hussein et al., 2016; Koneru et al., 2018; Calais et al., 2019; de Camargo et al., 2019; El Gabry et al., 2019; Habib et al., 2019; Heriot et al., 2019; Nesterovics et al., 2019; Płońska-Gościniak et al., 2019; San et al., 2019; Witten et al., 2019; Bohbot et al., 2021; Duval et al., 2021; Jędrzejczyk-Patej et al., 2021; Michałowska et al., 2021; Pyo et al., 2021).

Considering false culture-negative potential, when suspicion of IE remains then other techniques are emerging as additional diagnostic tools. This includes serological and molecular techniques, to include broad-range polymerase chain reaction (PCR) analysis, next-generation sequencing (Oberbach et al., 2017; Kolb et al., 2019; Zeng et al., 2022), and mass spectrometry (Holler et al., 2011; Singhal et al., 2015). At the same time, false negative PCR results have been reported with positive cultures (Gauduchon et al., 2003; Breitkopf et al., 2005) and identifying new isolates with mass spectroscopy relies on pre-existing databases input. Since limitations exist for all identification tools, a multimodal diagnostic approach for pathogen identification would be best in the modified Duke criteria.

### Imaging modalities

Transthoracic echocardiography (TTE) is the most common first-line imaging modality for IE and is used to visualize suspicious valvular insufficiencies, biofilms, and potential IE complications, such as abscess formation (Habib et al., 2015). Recent reports on the sensitivity of TTE is 84% for native valve, 63% for prosthetic valve, and 17% for cardiac devices (Table 2) (Cahill and Prendergast, 2016; Sivak et al., 2016; Sekar et al., 2017; Bin Abdulhak et al., 2018; de Camargo et al., 2019; Pettersson and Hussain, 2019; Slawinski et al., 2019; Horgan et al., 2020b; Rezar et al., 2021). This reduced sensitivity of prosthetic valves and intracardiac devices can be explained by imaging challenges, such as artifacts induced by metal components (Chambers and Bayer, 2020; Hubers et al., 2020). If TTE is negative and a high level of suspicion for IE persists, then transesophageal echocardiography (TEE) should also be performed (Sedgwick and Scalia, 2016; Sordelli et al., 2019; Hubers et al., 2020). TEE provides a better characterization of local abnormalities and can detect valvular biofilms as small as 1 mm, whereas biofilms smaller than 5 mm have a reduced TTE sensitivity of 25% (Erbel et al., 1988; Cuervo et al., 2021). As shown in Table 2, TEE has a sensitivity of 67% for cardiac devices and 94% for native valves, which is 50 and 10% higher than TTE respectively (Sekar et al., 2017; Doring et al., 2018; Koneru et al., 2018; Galar et al., 2019b; Ivanovic et al., 2019; Slawinski et al., 2019; Horgan et al., 2020b; Galea et al., 2020; Sifaoui et al., 2020; Aguilera et al., 2021; Jędrzejczyk-Patej et al., 2021). Nevertheless, the reported sensitivity of TEE for cardiac devices is limited. A systemic review also highlights the importance of considering the pre-disposing valvular intervention and the valve used, where echocardiography could only detect IE in 34% of patients that had previously undergone a transcatheter pulmonary valve implantation, also known as percutaneous pulmonary valve implantation, with the Melody valve (Abdelghani et al., 2018). As neither TTE and TEE are 100% sensitive and if positive blood cultures persist, intracardiac echocardiography (ICE) may be considered (Abdelghani et al., 2018; Blomström-Lundqvist et al., 2020). ICE probes are used to diagnose CDRIE and have high image resolution and can be oriented in any direction within the heart (Ali et al., 2011; Narducci et al., 2013). However, the role of ICE for NVE and PVE remains unclear, with limited supporting literature (Kolodner et al., 2007; Bouajila et al., 2017; Abdelghani et al., 2018; Yang et al., 2019) and cost/benefits need to be determined since it is more costly and invasive as well as not sustainable since each probe is single-use.

**TABLE 2:** Diagnostic accuracy with reported sensitivity and specificity of various imaging modalities.

Imaging	All IE		NVE		CDRIE		PVE	
	Se (%)	Sp (%)	Se (%)	Sp (%)	Se (%)	Sp (%)	Se (%)	Sp (%)
TTE	71 (60–82)	57^a^	84 (70–98)	93^a^	17^a^	100^a^	63 (60–65)	79 (67–95)
TEE	81 (36–95)	70 (42–85)	94 (91–96)	78 (67–88)	67^a^	100^a^	84 (78–91)	67 (57–75)
[^18^F]FDG PET/CT	81 (74–88)	86 (79–92)	36 (17–68)	97 (85–100)	82 (56–96)	90 (80–100)	91 (75–100)	67 (29–93)
WBC SPECT/CT	90 (86–100)	98 (95–100)	—	—	73 (60–84)	87 (74–100)	—	—
CT	53 (16–89)	84 (71–96)	49 (11–80)	77 (63–92)	75^a^	86^a^	73 (19–96)	78 (50–98)

a Only reported once since 2016.

CDRIE, cardiac device-related infective endocarditis; CT, computed tomography; [^18^F]FDG, 18F-fluorodeoxyglucose; IE, infective endocarditis; NVE, native valve endocarditis; PET, positron emission tomography; PVE, prosthetic valve endocarditis; Se, sensitivity; Sp, specificity; SPECT, single photon-emission computed tomography; TEE, transesophageal echocardiography; TTE, transthoracic echocardiography; WBC, white blood cell. The values in this graph were obtained using the following references: (Cahill and Prendergast, 2016; Wang A. et al., 2018; Bin Abdulhak et al., 2018; DeSimone and Sohail, 2018; Doring et al., 2018; Karchmer et al., 2018; Vincent and Otto, 2018; Galar et al., 2019a; Ivanovic et al., 2019; Pettersson and Hussain, 2019; Slawinski et al., 2019; Horgan et al., 2020b; Blomström-Lundqvist et al., 2020; Chambers and Bayer, 2020; Galea et al., 2020; Hubers et al., 2020; Aguilera et al., 2021; Jędrzejczyk-Patej et al., 2021; Rezar et al., 2021).

For CDRIE diagnosis, additional nuclear imaging techniques are recommended to increase the detection sensitivity from 56–88% to 78–98%, namely positron-emission tomography with 18F-fluorodeoxyglucose integrated with computed tomography ([^18^F]FDG PET/CT) or radiolabeled white blood cell (WBC) single photon-emission computed tomography (SPECT) (Cahill and Prendergast, 2016; Blomström-Lundqvist et al., 2020). This imaging is recommended when echocardiography is negative and blood cultures are positive (Blomström-Lundqvist et al., 2020). These additional nuclear imaging techniques should also be used in patients with a suspicion of PVE, and abnormal activity around the implantation site is considered a major diagnostic criterion (Habib et al., 2015). The sensitivity of [^18^F]FDG PET/CT is 15% higher than echocardiography for cardiac devices (6% for prosthetic valves), and 58% lower for native valves (Guenther et al., 2015; Dell’Aquila et al., 2016; Fagman et al., 2016; Granados et al., 2016; Memmott et al., 2016; Salomäki et al., 2017; Sánchez-Enrique et al., 2018; Calais et al., 2019; de Camargo et al., 2019; Abikhzer et al., 2020; Gomes et al., 2020; Wang et al., 2020). Besides the poor detection accuracy in NVE, this imaging modality has possible false-negatives in patients with small biofilms, possible false-positives concerning early post-surgical prosthetic valve implantation (Rosenbaum et al., 2006) or when active thrombi, cardiac tumors, and atrial fibrillation are present, a complex preparation protocol, and limited availability in peripheral centers (Marchetta et al., 2017). Current guidelines stipulate that [^18^F]FDG PET/CT should only be considered for IE diagnosis when > 3 months have elapsed after valve implantation to prevent false positives from artefacts following surgery (Habib et al., 2015). Radiolabeled WBC imaging with SPECT/CT improves detection and identification of endocardial involvement in IE by differentiating superficial surgical site infections from true generator pocket infections (Erba and Slart, 2020). In addition, this imaging modality has a sensitivity of 73% in CDRIE patients which can improve the risk stratification of patients with CDRIE (Małecka et al., 2018; Calais et al., 2019; Holcman et al., 2019). Positive results of WBC SPECT/CT scans are associated with an increased in-hospital mortality rate and complete hardware removal (Małecka et al., 2018; Calais et al., 2019; Holcman et al., 2019). Despite these promising features, this technique also has limitations, to include long scan-time, complex preparation procedure, lower spatial resolution compared with PET/CT, and limited availability for appropriate hospital-wide implementation (Erba and Slart, 2020).

Other imaging modalities, such as cardiac CT with or without angiography, can be used after a negative echocardiography to detect valvular complications, such as valvular regurgitation and paravalvular complications like abscesses or pseudoaneurysms (Vincent and Otto, 2018; Blomström-Lundqvist et al., 2020). CT can be advantageous because it has an improved spatial resolution compared to echocardiography that allows for the detection of infected valve manifestations, such as biofilms, leaflet thickening, valve perforation, valve aneurysm, and vascular complications like mycotic aneurysm, arterial emboli, and septic pulmonary infarcts (Vincent and Otto, 2018; Blomström-Lundqvist et al., 2020; Erba and Slart, 2020). However, Koneru et al. reported CT having a low sensitivity (16%) for all IE, and more specifically 11% in NVE and 19% in PVE, which is low compared to other papers which reported sensitivities of 89% for all IE, 57–80% in NVE, and 82–96% in PVE (Koneru et al., 2018; Hryniewiecki et al., 2019; Gomes et al., 2020; Sifaoui et al., 2020; Velangi et al., 2020; Michałowska et al., 2021). Overall, various imaging modalities fulfil an essential role in the evaluation and management of IE. A multimodality imaging approach in the evaluation of IE-suspected patients should be an early consideration to arrive at a definitive diagnosis.

## Causative microbes

**FIGURE 2:**
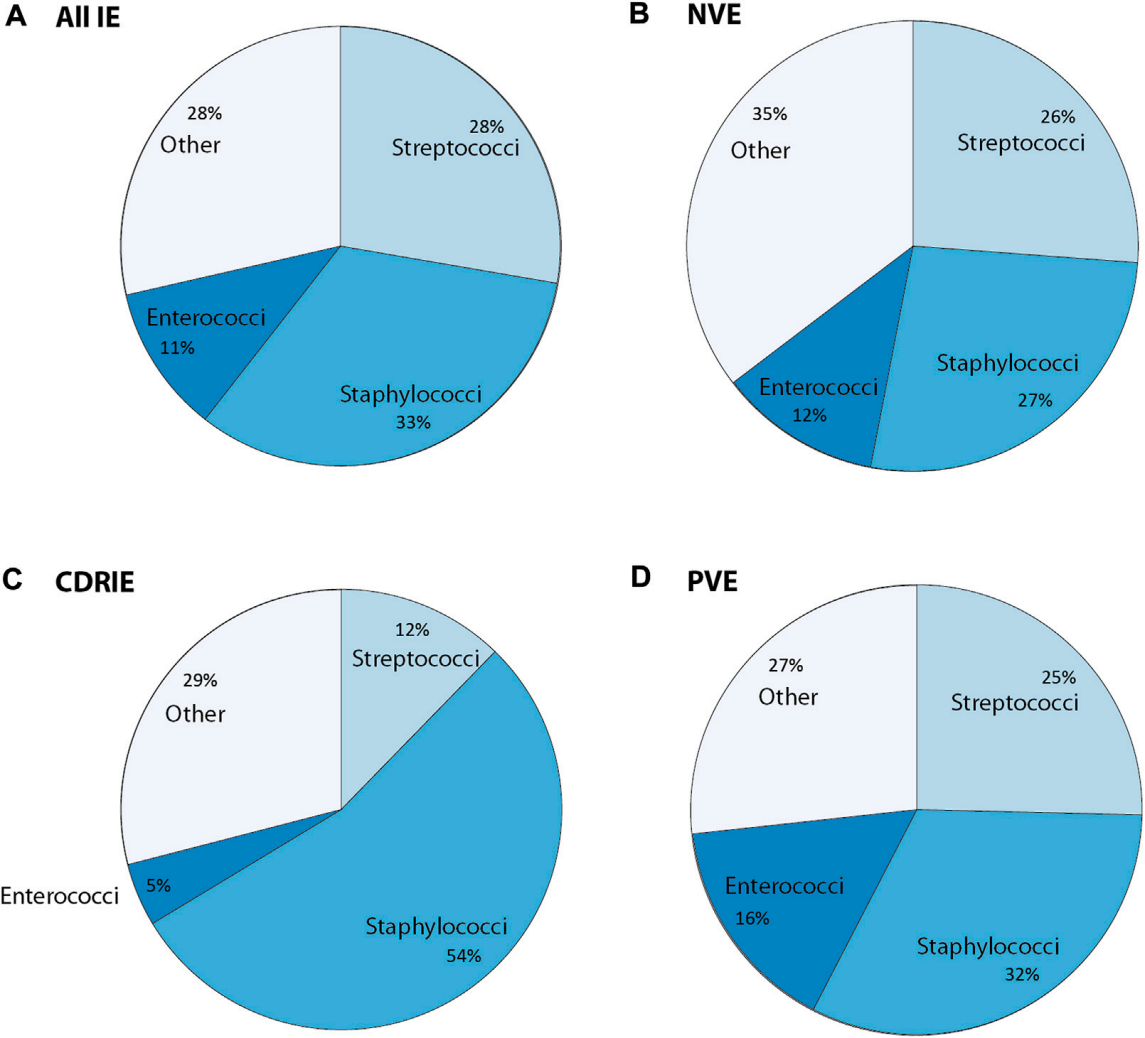
Overview of causative bacteria in infective endocarditis (IE) concerning **(A)** all IE cases, **(B)** native valve endocarditis (NVE), and **(C)** cardiac device related-infective endocarditis (CDRIE) that is further subdivided into **(D)** prosthetic valve endocarditis (PVE). Other refers to either other cultured or unidentified microorganisms. The values in this graph were obtained using the following references (Cahill and Prendergast, 2016; Wang A. et al., 2018; DeSimone and Sohail, 2018; DeSimone and Sohall, 2018, Babeș et al., 2021; Doring et al., 2018; Teoh and Hannan, 2018; Slawinski et al., 2019; Mateos Gaitán et al., 2020; Khalil and Soufi, 2022).

Staphylococci, streptococci, and enterococci bacterial species are estimated to account for ∼80% of all cases (Cahill and Prendergast, 2016; DeSimone et al., 2019; Chambers and Bayer, 2020). Microorganism prevalence was evaluated based on all IE, NVE, CDRIE, PVE (Figure 2). *Staphylococcus aureus* accounts for 27% of NVE, followed by streptococci (26%), and enterococci (12%) (de Camargo et al., 2019; El Gabry et al., 2019; Habib et al., 2019; Heriot et al., 2019; San et al., 2019; Chambers and Bayer, 2020; Hubers et al., 2020; Bohbot et al., 2021; Duval et al., 2021; Pyo et al., 2021). Coagulase-negative staphylococci have a prevalence of 6.3% in NVE, which is in contrast to CDRIE with 25.2% (Hussein et al., 2016; Koneru et al., 2018; Calais et al., 2019; Habib et al., 2019; Nesterovics et al., 2019; San et al., 2019; Witten et al., 2019; Bohbot et al., 2021; Duval et al., 2021; Pyo et al., 2021). Microorganisms obtained from cultures of CDRIE patients were predominately staphylococci (54%), followed by streptococci (12%) and enterococci (5%) (Habib et al., 2015; Hussein et al., 2016; Koneru et al., 2018; Calais et al., 2019; Nesterovics et al., 2019; Płońska-Gościniak et al., 2019; Witten et al., 2019; Jędrzejczyk-Patej et al., 2021). Staphylococci are considerably more prevalent in CDRIE compared to NVE (27%) and PVE (32%) (Hussein et al., 2016; Koneru et al., 2018; Calais et al., 2019; de Camargo et al., 2019; El Gabry et al., 2019; Habib et al., 2019; Heriot et al., 2019; Nesterovics et al., 2019; Płońska-Gościniak et al., 2019; San et al., 2019; Witten et al., 2019; Bohbot et al., 2021; Duval et al., 2021; Jędrzejczyk-Patej et al., 2021; Michałowska et al., 2021; Pyo et al., 2021). Streptococci (25%) and enterococci (16%) also cause PVE (de Camargo et al., 2019; Habib et al., 2019; San et al., 2019; Duval et al., 2021; Michałowska et al., 2021; Pyo et al., 2021). Enterococci causing NVE and CDRIE tends to be more common in elderly or chronically ill patients (Cahill and Prendergast, 2016; Yang and Frazee, 2018). The elderly population and patients who undergo transcatheter aortic valve implantations, also known as percutaneous aortic valve implantation, are most at risk for enterococcal infections, particularly in combination with pre-existing comorbidities (Munita et al., 2012; DeSimone et al., 2021). For IE caused by enterococci, there has been an increasing temporal trend of approximately 5% per year (DeSimone et al., 2021).

Although less common, other microorganisms can be the cause of IE. This includes, though not limited to, *Haemophilus*, *Aggregatibacter*, *Cardiobacterium*, *Eikenella*, and *Kingella* species known collectively as HACEK organisms, as well as *Coxiella burnetii*, aerobic gram-negative bacilli, and fungi (Chambers and Bayer, 2020; Hubers et al., 2020). HACEK are gram-negative bacteria and fastidious, i.e. difficult to culture (Wang F. et al., 2018). Fungal endocarditis is rare for both native valves and cardiac devices, and is associated more with CDRIE, particularly PVE (Ojha and Dhamoon, 2022). *Candida* and Aspergillus species are the primary causative fungal microbes, where *Candida albicans* is the most common overall (Shokohi et al., 2014; Yuan, 2016) and Aspergillus species more prevalent in PVE (Pasqualotto and Denning, 2006). Fungal endocarditis is extremely challenging to diagnosis and thus start appropriate treatment as it presents similarly to bacterial endocarditis (Wang F. et al., 2018; Ammannaya and Sripad, 2019), with 82% receiving a delayed or mistaken diagnosis, 28–77% diagnosed only post-mortem, and a mortality rate of 72% (Seelig et al., 1974; Ellis et al., 2001; Yuan, 2016).

## Treatment and outcomes

Management of IE patients consists largely of antimicrobial therapy and surgery. Without treatment, IE is considered to be fatal. Treatment focus is placed on preventing the development of complications, such as embolization and heart failure as well as progression from a local to a systemic infection (i.e., sepsis and septic shock) which has a four-fold increase in mortality risk (Werdan et al., 2014).

### Antimicrobial therapy

Antimicrobial therapy is required in the management of IE focusing on antibiotics for a prolonged period. The choice of antibiotic and the duration of administration itself is based on the causative pathogen, potential antibiotic resistance, and type of infected material, whether native tissue, prosthetic valve, or cardiac device (Wang F. et al., 2018). Antimicrobial treatment of IE is extensively described in the European Society of Cardiology (ESC), American Heart Association (AHA), and Stichting Werkgroep Antibioticabeleid (SWAB) guidelines (Habib et al., 2015; Baddour et al., 2015, T.W. van der Vaart, 2019).

Rifampicin use remains controversial and is associated with diverse adverse effects, including hepatotoxicity, nephrotoxicity, and high-risk of drug interactions (Almatrafi et al., 2021). Studies have shown that rifampicin did not have a significant effect on mortality or bacteremia duration in patients with a *S. aureus* infection (not only IE) (Thwaites et al., 2018), and specifically for PVE with *S. aureus* rifampicin did not reduce mortality and those patients had longer hospital stays (Le Bot et al., 2021). However, rifampicin did lead to lower disease recurrence (Thwaites et al., 2018). In another study, the addition of gentamycin to vancomycin and rifampicin also did not result in lower mortality and the occurrence of new renal failure or worsening of previous failure was high both in patients treated with and without gentamicin (Ramos-Martínez et al., 2018). Worse outcomes could potentially be attributed to comorbidities and infection severity (Goenaga Sánchez et al., 2017). Regardless, additional clinical evidence is necessary so a definitive decision can be derived on if rifampicin and/or gentamycin should continue to be added to the treatment of PVE patients with *S. aureus* (Di Domenico et al., 2019).

Another controversial topic in IE empirical treatment is antibiotic prophylaxis. The incidence of IE is increasing and although this is likely multifactorial, it has been suggested that the recent increases can be attributed to the change in prophylaxis guidelines (Pant et al., 2015). To prevent IE during at-risk procedures, such as invasive dental procedures and implantable cardiac electronic device insertion, international guidelines changed from the routine use of a single-administration of an antibiotic shortly before procedures to only used in patients with a high-risk for poor IE outcomes. This patient population includes those having prosthetic or repaired valves, a previous IE diagnosis, or congenital heart disease (Wilson et al., 2007; Habib et al., 2009; Habib et al., 2015). The correlation between antibiotic prophylaxis and the incidence of IE before and after the updated AHA guidelines of 2007 was investigated (Thornhill et al., 2018). Patients were categorized based on their low, moderate, and high risk of developing IE. Data acquired from May 2003 to August 2015 revealed no increase of IE cases in the low-risk patient group. However, for the high- and moderate-risk patients groups increases were observed. Recently, a significant association between the use of prophylaxis and reduced IE incidence following invasive dental procedures (particularly extractions and oral-surgical procedures) was found (Thornhill et al., 2022). These data support AHA, ESC, and other guideline recommendations that prophylaxis before invasive dental procedures should continue for individuals with a high IE risk. In addition to prophylactic antibiotics, educating patients on the importance of good dental and skin hygiene and sterility during invasive procedures remain essential IE prevention methods. It should be noted that good hygiene is not only more difficult to achieve in developing countries, also the awareness of its importance in preventing IE has been reported to be inadequate not only in the general population, but also among general practitioners and specialists within the fields of cardiology (Maharaj and Parrish, 2012).

### Surgery

When antimicrobial therapy fails, or other complications arise due to IE such as valve leakage or abscess, these patients often require surgery. At least 50% of IE patients are estimated to undergo surgery during hospitalization, and when stratified for tissue or device this is 54% for NVE, 46% for PVE, and 65–95% for CDRIE (Ortiz-Bautista et al., 2017; Habib et al., 2019; Roder et al., 2020). Surgery can be required however also cannot be a treatment option due to patient refusal, high surgical risk, neurological complications, or death before surgery (Ortiz-Bautista et al., 2017; Habib et al., 2019). In-hospital mortality following surgery was highest for PVE (up to 27%) when compared to NVE (16%) and CDRIE (8–15%) (Ortiz-Bautista et al., 2017; Habib et al., 2019; Nasso et al., 2021).

Surgery for NVE is generally performed due to valvular dysfunction or rupture, uncontrolled IE, heart failure, abscess, sepsis, as well as to prevent embolization (El Gabry et al., 2019; Chambers and Bayer, 2020; Pyo et al., 2021) and generally consists of valve replacement or repair, with valve replacement more frequent (Said et al., 2018; Witten et al., 2019; Park et al., 2020). Concerning NVE surgical heart procedures, bioprostheses (35–40%) were utilized most, followed by mechanical prostheses (25–39%), repair (15–31%), and homograft placement (4–11%) (Said et al., 2018). The 1-year mortality rate following surgery for NVE is 4–22%, and a 5-year survival rate reported as 77% (El Gabry et al., 2019; Defauw et al., 2020; Park et al., 2020; Pyo et al., 2021; Weber et al., 2021). NVE comparative studies observed a higher 30-day mortality after valve replacement (8–17%) compared to valve repair (4–14%) (Toyoda et al., 2017; Defauw et al., 2020). Surgery for PVE is indicated for mobile or larger than 10 mm biofilms, heart failure, valvular dysfunction, abscess, persistent sepsis, acute renal failure, and evidence or high risk of embolic events (Pyo et al., 2021). Aortic valve procedures are performed most often (72%), followed by mitral (39%), tricuspid (9%) and pulmonary (3%) valves (Habib et al., 2019). Adverse outcomes can occur in PVE surgical management, which include embolization (21%), acute renal failure (21%), new-onset dialysis (20%), stroke (13–19%), reoperation for bleeding (12–14%), low cardiac outcome syndrome (13%), heart failure (11%), and persistent fever (13%) (Pyo et al., 2021; Weber et al., 2021). Additionally, fungal endocarditis can occur up to 3 years following surgery (Shokohi et al., 2014). The post-surgical 30-day mortality for PVE is 14–20% and at 1-year 22–36% (Pyo et al., 2021; Weber et al., 2021), with 27% in-hospital mortality in patients with a prior aortic valve replacement (Nasso et al., 2021). For both NVE and PVE, the type of prosthesis (biological or mechanical) chosen is not associated with mortality while (longer) aortic cross-clamp times are a significant mortality predictor (Nasso et al., 2021).

Current valve replacement interventions can either be surgical or transcatheter. Transcatheter interventions were only first approved by the FDA in 2012 for high-risk patients. Comparing surgical and transcatheter aortic valve replacement in England over 10 years, the surgical cohort had a higher IE incidence rate of 4.8 (2.4% cumulative) compared to 3.6 (1.5% cumulative) in the transcatheter cohort (Cahill et al., 2022). This significant higher IE risk was also found in another study (Lanz et al., 2021), however these two studies are in contrast to several others that found no significant differences (Kolte et al., 2018; Butt et al., 2019; Noriaki et al., 2019; Summers et al., 2019; Fauchier et al., 2020). For transcatheter pulmonary valve implantation, two valves have been approved: the Edwards Sapien valve and Medtronic Melody valve. The cumulative incidence of IE concerning the Melody valve was 3–25%, with 59% of cases occurring within the first 2 years after implantation (Abdelghani et al., 2018). In comparative and meta-analysis studies, the Sapien valve was found to have a significantly decreased cumulative IE incidence at 0–1% compared to the Melody valve at 5–24% (Hascoet et al., 2017; Lehner et al., 2019). In a single-center study in Munich, Germany, the incidence of IE was three times higher for the Melody valve (1.6%) compared to the surgical pulmonary valve replacement group (0.5%), however the estimated survival between the two groups did not significantly differ (Georgiev et al., 2020).

Most cases of CDRIE managed by surgery underwent surgical device removal (95%) (Ortiz-Bautista et al., 2017; Habib et al., 2019; Roder et al., 2020; Jędrzejczyk-Patej et al., 2021). Adverse effects of device removal include acute renal failure (21%), shock (15%), embolization (13%), and persistent fever (12%) (Habib et al., 2019). For implicated cardiac device drivelines, initial surgical management can include drainage and dead tissue debridement. When stratified for pacemakers and implantable cardioverter-defibrillators, 47–53% patients underwent percutaneous lead extraction, 41–53% surgical lead extraction, and 5% percutaneous catheter extraction (Ortiz-Bautista et al., 2017; Habib et al., 2019; Roder et al., 2020; Jędrzejczyk-Patej et al., 2021). Device reimplantation occurs once a negative blood-culture is obtained, which in 77% of patients was after a median time of 13 days (Ortiz-Bautista et al., 2017). In a 13-year Danish nationwide study, following reimplantation, the mortality rate was 42% for pacemakers and 21% for cardioverter-defibrillators (Özcan et al., 2017), which is higher than the overall in-hospital mortality rate of CDRIE (Ortiz-Bautista et al., 2017; Habib et al., 2019).

Early surgery for IE patients, defined as emergency or within 48 h once an indication for surgery is established (Pettersson and Hussain, 2019), have improved outcomes without increasing the incidence of postoperative neurological complications (Samura et al., 2019) and is associated with lower in-hospital stroke rates and length-of-stays compared to late surgery (Kousa et al., 2020). For CDRIE, delayed extraction may also contribute to higher mortality (Kim et al., 2014). Following guidelines, the timing of surgery should be without delay after definitive diagnosis for NVE, PVE, and CDRIE (Pettersson and Hussain, 2019; Blomström-Lundqvist et al., 2020). However, there are conflicting studies that observed no difference in neurological outcomes and mortality between early and late surgery (Oh et al., 2016; Okita et al., 2016; Huuskonen et al., 2019; Kousa et al., 2020). Huuskonen et al. stated that patients receiving early surgery had a higher reoperation rate and greater recurrent mitral regurgitation (Huuskonen et al., 2019). Furthermore, early surgery has also been associated with a trend toward higher 6-month overall mortality compared with later surgery during hospitalization (Wang et al., 2019) and had higher rates of postoperative bleeding and pericardial effusion (Kousa et al., 2020). This could be due to earlier operated patients being significantly sicker at the time of surgery with a higher potential to have frail tissue due to active infection (Huuskonen et al., 2019). Surgical decision and timing are important yet challenging factors in the management of IE and thus, should be deliberated as early as possible by a multispecialty clinical team that can consider factors such as comorbidities and neurological complications with a greater knowledge depth (Pettersson and Hussain, 2019; Habib et al., 2015; Wahadat et al., 2022).

## Investigative research approaches

**FIGURE 3:**
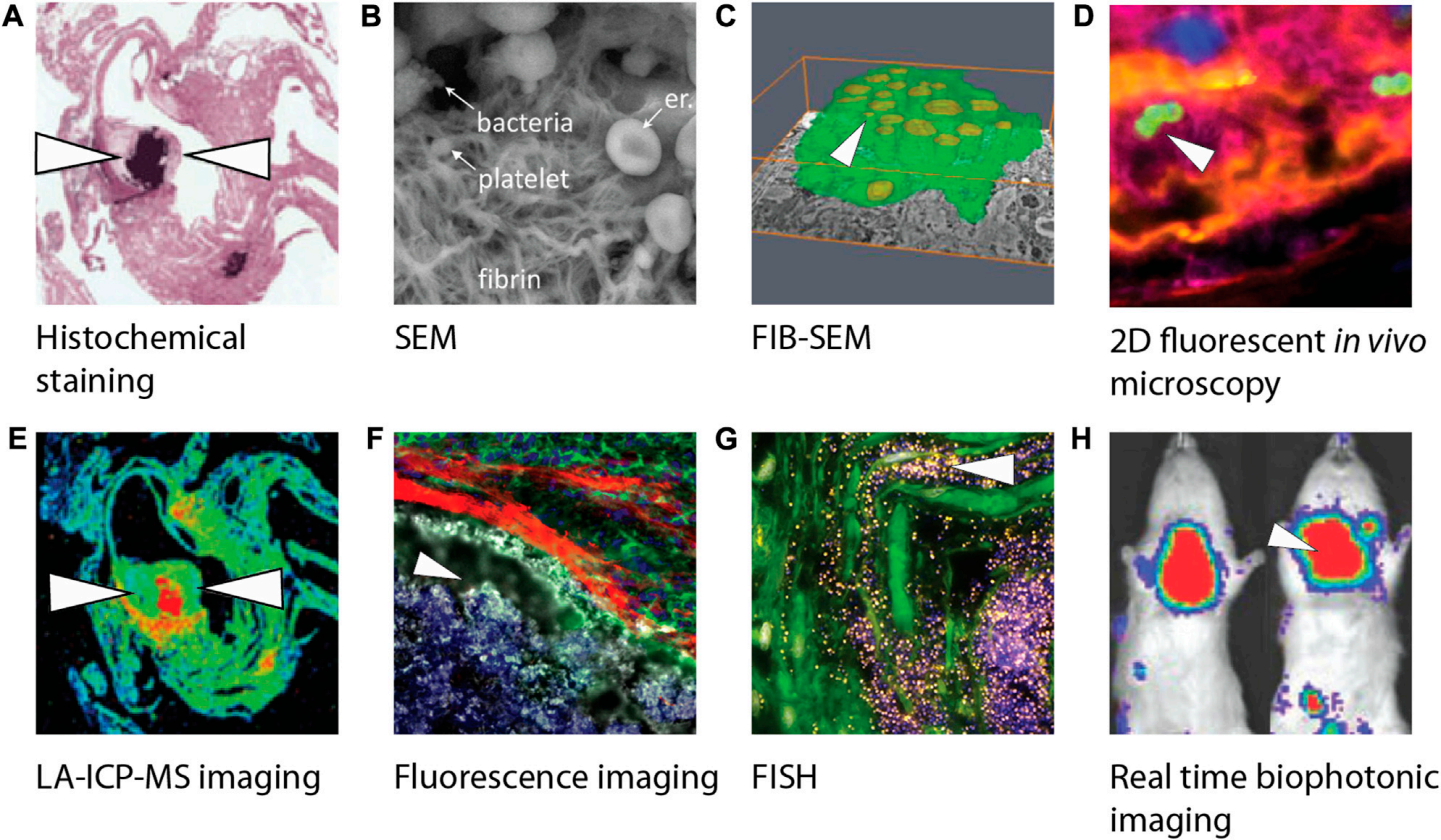
Overview of different biofilm visualization modalities used in infective endocarditis research. **(A)** Crystal violet staining visualizing gram-positive bacteria of paraffin-embedded female C57BL/6 mice heart tissue slices using light microscopy (Schwarz et al., 2022). **(B)** SEM image visualizing bacteria including a platelet-containing fibrin network (Hannachi et al., 2020). **(C)** Three-dimensional reconstruction of phagocytic cells (green) infected by bacteria (yellow) using FIB-SEM (Oberbach et al., 2017). **(D)** Confocal image of an early infective endocarditis lesions in mice visualizing platelets (pink), fibrin (yellow), endothelium (blue) and *S. aureus* (green) (Liesenborghs et al., 2019). **(E)** LA-ICP-MS imaging makes it possible to produce a quantitative distribution map (min to max, blue to red) of elemental distribution (Ca, Mg, Mn, Fe, Cu, or Zn) within female C57BL/6 mice heart tissue infected by bacteria (Schwarz et al., 2022). **(F)** Visualization of myeloid cells’ (green) inability to interact with bacteria (white) due to a fibrin barrier (red) in endocarditis in mice (Panizzi et al., 2020). **(G)** Visualization of *S. epidermidis* on aortic valve tissue with bacteria after incubation with a FISH probe (yellow) and DAPI (blue) (Lauten et al., 2021). **(H)** Real time imaging of rats infected by *S. aureus* using a bioluminescent *in vivo* imaging system (Xiong et al., 2005) showing high (red) to low (blue) bioluminescent signals. White arrowheads indicate the presence of bacteria. Images were adapted with permission from the original publishers and used as examples. SEM, Scanning electron microcopy; FIB-SEM, Focused ion beam scanning electron microscopy; LA-ICP-MS, Laser ablation induction coupled plasma mass spectrometry; FISH, Fluorescence *in situ* hybridization; DAPI, 4′,6-diamidino-2-phenylindole.

This part of the review is focused on the research techniques used to improve the understanding of IE pathogenesis and address the diagnostic and treatment challenges to improve or develop innovative strategies. Various techniques are utilized to investigate IE, as seen in Figure 3, and are thus discussed, including histopathology, microscopy, metagenomics, proteomics, and others.

### Histopathological analysis

Histological staining of surgically excised cardiac tissue and valves in connection with IE, whether definitive or suspected IE, has been routinely performed for decades (Figure 3A). The subsequent histopathological analysis is used to confirm diagnosis or provide support for alternative disease. One study compiling the histopathological results of over 800 valves with definite IE (59% NVE) found 92% having evidence of inflammation and/or microorganisms (Ely et al., 2016). Not one single histological finding achieved this 92% accuracy, with 67% samples showing microorganisms, 62% chronic inflammation markers (neovascularization, lymphocytes, histiocytes, foamy histiocytes, or giant cells), and 56% acute inflammation marker (polymorphonuclear leukocytes). Fibrin deposits, which are implicated in the pathogenesis of IE, were found in 61% of cases and clustered together with microorganism presence. No significant connections were made between valve type, valve location, and microorganism presence.

Histopathological analysis is also performed in conjunction with CDRIE, however to a lesser extent given that mechanical devices themselves cannot be histologically processed. Lead-associated intracardiac masses in connection with positive blood cultures (*S. aureus*, *S. epidermidis*, and Propionibacterium species) that were histologically investigated were found to be primarily composed of fresh fibrin with inflammatory cell infiltration, mostly neutrophils (Chang et al., 2019; Miyagi et al., 2020). Although bacteria were not found histologically, the primary findings still support that intracardiac masses can be related to CDRIE since extracted masses without positive blood cultures only showed thickened endocardium and collagenous tissue (Miyagi et al., 2020). Biopsies of intracardiac masses obtained using a triple-loop wire snare (Salaun et al., 2017) or a bioptome with a steerable sheath (Chang et al., 2019) with access *via* the femoral vein, demonstrated their diagnostic value resulting in either the continuation or discontinuation of antibiotic therapy. Thus, biopsies of these masses for histopathological analysis should be considered during diagnosis to allow for accurate differentiation between thrombus and biofilm for correct and prompt treatment.

There are limitations to histopathological analysis, namely intra-inter pathologist variability and entire excised valves are not analyzed due to procedural and analytical tool constraints. 2D tissue sections of only a few micrometers thick of samples that can easily be larger than 10 mm can result in missing infection-indicating information, especially given the complexity and heterogenous make-up of IE biofilms. 3D histology techniques are in development and show histopathological promise (Eberle et al., 2014), though have yet to be evaluated for IE.

### Microscopic evaluation and imaging

**FIGURE 4:**
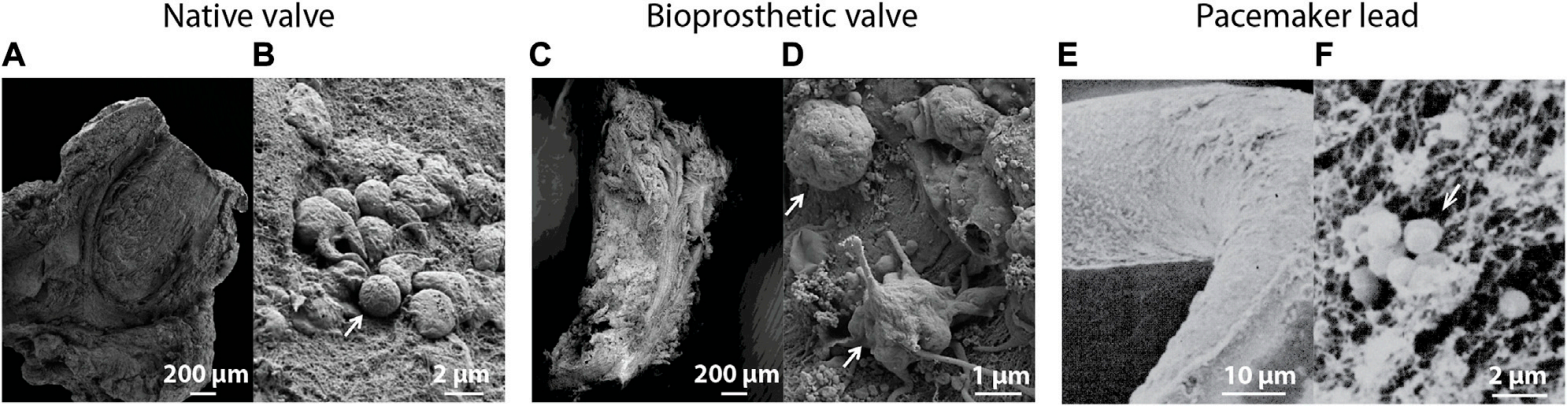
Scanning electron microscopic images of **(A)** an infected native heart valve with **(B)** a corresponding magnified image showing intact bacteria on the tissue surface (Oberbach et al., 2017). **(C)** Overview of an infected biological prosthetic valve with **(D)** a corresponding magnified image showing intact bacteria surrounded by a fibrous surface (Oberbach et al., 2017). **(E)** Visualization of a biofilm on the surface of a pacemaker lead with **(F)** a corresponding magnified image showing bacteria surrounded by a fibrillar substrate (Marrie and Costerton, 1984). White arrows indicate the presence of bacteria. Images were adapted with permission from the original publishers and used as examples.

The adhesion and spatial orientation of bacterial biofilms can be observed with scanning electron microscopy (Litzler et al., 2007) (Figures 3B, 4). Additionally, it can be used to determine the efficiency of antibiotic treatment on biofilms cultured on different materials used for prosthetic implants and other medical devices, while simultaneously evaluating the matrix produced by the pathogen (Jahanbakhsh et al., 2020). Oberbach et al. aimed with their research to identify the biodiversity of bacteria species in three infected native and five prosthetic heart valves (Oberbach et al., 2017). Focused ion beam scanning electron microscopy (FIB-SEM) was used to investigate the micro-environment of the infected heart valves, and localization and distribution patterns of the causative bacteria were analyzed. FIB-SEM reconstructions visualized the intracellular and intramural localization of bacteria, which might contribute to the sensitivity of culture-based diagnostic characterization of bacteria causing IE (Figure 3C).

Fluorescence *in situ* hybridization (FISH) is a relatively new technique that can be used for the identification, location and spatial organization and activity of the pathogen causing IE by using oligonucleotides (specific sequences of RNA or DNA) (Lauten et al., 2021). However, it remains difficult to implement FISH as a diagnostic tool due to the need of technical and medical expertise as well as the lack of diagnostic quality control (Kikhney and Moter, 2021). In research, FISH can broaden the understanding of IE pathogenesis. For example, Lauten et al. created a pulsatile two-chamber circulation model to grow biofilms on porcine heart valves under physiological conditions. Microscopic evaluation of fixated samples was carried out using several FISH probes (Figure 3G). Results showed metabolic active bacterial formations on the heart valve. Similar colonization patterns were observed between multiple samples. This *in vitro* infected heart valve model was compared to clinical IE samples and showed good comparability with these biofilms. Furthermore, the use of FISH with peptide nucleic acid probes (PNA) has previously been reported for the detection of *Coxiella burnetii* in heart valves and thrombi collected from patients with IE (Prudent et al., 2018). Compared to FISH using oligonucleotides, PNA FISH showed a higher sensitivity and specificity in clinical specimens (Prudent et al., 2018).

Although FISH can be used for the detection and characterization of bacteria in tissue sections, the manual assessment process can be time consuming. While tissue samples with fluorescent signal due to the binding of FISH probes can quickly be labeled as positive, a negative diagnosis requires a specialist to investigate the rest of the sample. Automated imaging analysis might be used for more efficient pathogen detection and aid specialists in identifying potential infected regions (Bruns et al., 2021).

Injected bacteria can be fluorescently labeled for detection using confocal microscopy post-mortem, or genetically modified by gene knock-out technology to investigate the function of specific genes in biofilm formation (Liesenborghs et al., 2019; Martini et al., 2020) (Figure 3D). The progression of the infection can also be monitored using cardiac magnetic resonance imaging, which Schwarz et al. used to reveal pronounced valve thickening, hypo-intensities, and masses on the heart valves of C57BL/6 mice within 24 h after *S. aureus* infection (Schwarz et al., 2021). Xiong et al. made use of an aortic IE rat model in combination with an engineered *S. aureus* isolate capable of bioluminescence (Figure 3H) (Xiong et al., 2005). Animals can serve as their own control without the need for sequential sacrifices at multiple timepoints*.*

Laser ablation inductivity coupled plasma mass spectrometry (LA-ICP-MS) was used on tissue slices of a heart from an IE mouse model (Schwarz et al., 2022) (Figure 3E). Three *S. aureus* isolates with specific adhesion deficiencies were used to investigate strain-specific patterns and were compared to sterile inflammation, and control samples. Element specific accumulations were quantified, and its distribution was compared between the experimental groups. Increased concentrations of calcium, magnesium, and zinc were observed in IE samples. LA-ICP-MS could distinguish between inflammation and infection and could be beneficial for characterizing tissue biopsies from patients suspected of IE.

### Clinical metagenomics

Although culture-based methods are generally accepted as the gold standard for pathogen identification, a proper readout requires 24–72 h and can be inconclusive or give false negative results (2.5–31%) (Brouqui and Raoult, 2001). Clinical metagenomics is an emerging, culture-independent approach utilizing nucleic acid sequencing that can identify infecting pathogens relatively quick (as quick as 6 h) with a high sensitivity (up to 96.6%) and specificity (up to 99%) (d'Humieres et al., 2021). Considering these advantages, proposals have been made for metagenomic techniques to be added as a routine diagnostic tool to the Modified Duke’s criteria (Millar et al., 2001; Tak and Shukla, 2004).

Several metagenomic techniques exist. A broad-range PCR technique that can be combined with next generation sequencing called 16S rDNA or 16S rRNA analysis has been successfully used for bacterial detection (Oberbach et al., 2017; Boujelben et al., 2018; Armstrong et al., 2021). Currently, PCR in IE can only be used to determine which bacteria are on valve- and other infected tissue retrieved after surgical intervention. Thus, this approach to determine the causative bacteria cannot be used for patients who do not require surgical removal of the infected heart valves. Identification of the causative pathogen in blood samples by PCR is more challenging, possibly due to a low concentration of microorganism in the samples (Vollmer et al., 2010). A thorough review about using PCR to detect microorganisms in IE has been written by Faraji et al. (Faraji et al., 2018).

To identify the causative pathogen in culture-negative patients not requiring valve surgery, To et al. used cell-free plasma metagenomic next-generation sequencing (mNGS) to detect pathogens in cell-free plasma of pediatric IE patients (To et al., 2021). Despite prolonged antibiotic treatment, the causative pathogen was successfully identified in eight out of the ten subjects. Since excised tissue is no longer a requirement for this technique, the disadvantage of broad-range PCR techniques requiring valve tissue is resolved. Another study used mNGS to retrospectively analyze 49 IE patients (43 patients with NVE and nine patients with PVE), of which 28 (57.1%) had positive blood or valve cultures. The remaining 21 patients (42.9%) had negative culture results. For all patients, including culture-negative IE patients, receiving empirical antibiotic treatment, the causative IE pathogen could be detected with mNGS (Cai et al., 2021).

Similarly, Eichenberger et al. used microbial cell-free DNA (mcfDNA) extracted from plasma obtained from patients to detect IE (Eichenberger et al., 2022). Although the sensitivity of mcfDNA was comparable to blood cultures (both 87%), the duration of detection after antibiotic treatment was estimated to be 38.1 days compared to 3.7 days positive blood cultures. After surgical intervention, mcfDNA was declined rapidly and may potentially be used as a marker for IE infection burden. Another technique to detect the causative pathogen in IE is nanopore sequencing. Unlike PCR, this technique does not require the amplification of DNA or RNA before sequencing. Cheng et al. used NGS and nanopore sequencing to detect the causative pathogen and their resistance genes in several culture-negative IE patients (Cheng et al., 2018). With each sequencing technique, the researchers were able to identify the pathogens in excised infected valve tissue.

Lilje et al. used whole genome sequencing (WGS) in combination with genome-wide association studies (GWAS: SNP and k-mer analysis) to search for genetic differences between bacterial *S. aureus* isolates from patients with IE and patient with bacteremia. When comparing the clonal complexes (CCs) between the two groups, no significant association of specific CCs was found (Lilje et al., 2017). Also, SNP analysis could neither highlight any statistical overrepresentation in the IE or bacteremia patient isolates group, nor after k-mer analysis of all IE and bacteremia samples (Lilje et al., 2017).

Metagenomic analysis is projected to be used more routinely in the future to identify unusual or fastidious pathogens (bacterial, fungal or viral) due to its increased sensitivity compared to contemporary diagnostic modalities. However, of all sequencing data, approximately 5% is usable for the identification of pathogens (Cheng et al., 2018). Considering that the remaining 95% of the generated data can be accounted for by patient material, methods to reduce this percentage could further improve pathogen detection to aid in a more rapid and accurate antimicrobial IE treatment.

### Proteomic analysis

Biofilms consist out of a plethora of different components, such as extracellular matrix components like fibrin and collagen, platelets, host immune cells and proteins produced by the pathogen. Mass spectrometry (MS) techniques can measure the presence of thousands of peptides in a single sample, which after bioinformatic analysis, can be assigned to specific proteins produced by the host or the causative pathogen. Martin et al. extensively described the proteome of native and bioprosthetic heart valve biofilms obtained from the clinic and noted a high abundance of fibrin and platelets in these biofilms (Martin et al., 2020). Of all proteins contributing to the biofilm (five on native heart valves, two on bioprosthetic heart valves and one from non-valvular complex IE), the 15 most abundant proteins of all examined biofilms made up for 57% of the samples proteomes and was similar between staphylococcal and non-staphylococcal biofilms. However, comparing the shared proteins in any combination of the non-staphylococcal biofilms (including both NVE and PVE) resulted in an average of 21% similarity, which is considerably less compared to the 56% shared proteins in staphylococcal biofilms. Furthermore, the amount of peptides overlapping between staphylococcal and non-staphylococcal biofilms was as high as 82%, which could be mostly explained by the contribution of host proteins from blood and neutrophils. New insights in the composition and formation of these biofilms might lead to new treatment opportunities and prospective biomarkers.

Different methods can also be combined for the identification of IE. Brinkman et al. used electro spray ionization MS on obtained PCR products for the identification of pathogens in formalin-fixed paraffin-embedded heart valves. This technique was used in 83 cases of IE, of which 59 with NVE and 24 with PVE. This approach allowed for the correct detection of antibiotic resistance genes (mecA in staphylococcal IE and vanA/B in enterococcal IE) in all IE cases in which the susceptibility towards antimicrobials was known, while also identify the causative pathogen in 55% concordant to microbiology (Brinkman et al., 2013). In 34% no identification of the causative pathogen was possible, which could potentially be attributed to degradation of DNA during histological sample treatment with formalin, degrading DNA in its process (Srinivasan et al., 2002).

Currently the detection of IE biomarkers is not being used in the clinic. Biomarkers indicating the presence of IE could be of great value for early diagnosis and several mass spectrometry approaches are aiming to detect IE by finding potential biomarkers (Martin et al., 2020; Snipsoyr et al., 2020). One such biomarker is Osteoprotegerin, which could potentially be used to exclude IE in patients suspected to have IE (Snipsoyr et al., 2020). A major challenge is the verification of possible biomarkers due to inter-patient variability and the low sensitivity and specificity in blood samples, which can be attributed to high abundance of blood protein peptides. These highly abundant peptides may conceal the presence of peptides from low abundant proteins, which is often the case for biomarkers. This stresses the importance of finding optimal sample preparation and bioinformatic analysis techniques.

### *In vitro, ex vivo,* and *in vivo* IE models

*In vitro* biofilm models have a wide-range of utility for infection research, such as high throughput screening of new drug treatments (Harrison et al., 2010), or for investigating (a-)synergism of antibiotics when combined with other therapies (Nair et al., 2016). However, infection models are often not developed specifically for IE. Although useful for initial experiments, *in vitro*, *ex vivo*, *and in vivo* biofilm-related models without an IE focus are not included in this review and the reader is referred to other extensive reviews (Coenye and Nelis, 2010; Lebeaux et al., 2013). Additionally, NVE model history and results are discussed at length by Lerche et al. (Lerche et al., 2021). This subsection will thus touch upon new literature as well as PVE and CDRIE and discuss controversies and considerations in IE model research.

To resemble early pathogenesis of IE, Lattwein et al. produced an *in vitro* infected blood clot model comprised out of human whole-blood clots retracted around silk sutures (Lattwein et al., 2018). Sterile clots were inoculated with a clinical *S. aureus* isolate. Blood clots were used to simulate the cardiac micro-thrombi to which bacteria adhere to before biofilm formation to better represent IE biofilms found in patients. After incubation, infected clots were placed in an flow system for treatment experiments.

A main disadvantage of *in vitro* models is the lack of a host immune system or blood pool proteins, resulting in a more artificial, non-representative IE biofilm model. Schwartz et al. aimed to create an *in vitro* NVE biofilm model which did not have this drawback (Schwartz et al., 2021). Their organoid-like model contained a leukocyte and platelet-rich fibrin patch system, which normally is applied to chronic wounds for treatment. This patch system thus represented damaged endothelium as a matrix for bacterial colonization. After formation of bacterial microcolonies by *S. aureus*, *Enterococcus faecalis* and *Streptococcus mitis*, increased antibiotic tolerance for all three pathogens was observed compared to its planktonic counterpart highlighting the importance of bacteria to form biofilms. The addition of host immune cells in this *in vitro model* is novel compared to other *in vitro* IE models and might in the future better predict personalized IE treatment outcomes.

IE model research has been performed focusing on the prevention of PVE and CDRIE after the implantation of prosthetic heart valves, catheter-associated biofilms and implantable cardiac devices (Litzler et al., 2007; Abdelhady et al., 2013). Litzler et al. investigated the adhesion of *S. aureus*, *S. epidermidis*, and *Pseudomonas aeruginosa* to three pyrolytic carbon mechanical heart valves, with and without silicon, using bioreactors (Litzler et al., 2007). The hydrophobicity, roughness, surface chemistry, electrostatic forces and surface free energy all seem to influence the adhesion of bacteria to the mechanical heart valves. The importance of the surface chemistry of biomaterials on biofilm formation has also been further investigated by MacKintosh et al. (MacKintosh et al., 2006). They highlighted the importance of using the correct media in *in vitro* set-ups. In their study, the use of serum promoted the adhesion and aggregation of *S. epidermidis* to charged surfaces. An *ex vivo* IE model using freshly excised porcine heart valve punch biopsies to study *Enterococcus faecalis* adherence has also been developed (Chuang-Smith et al., 2010). One major advantage of investigating bacterial adherence in *in vitro* and *ex vivo* set-ups, are the controlled environment and reproducibility of conditions, which is inherently less for clinical studies due to inter-individual differences.

Several factors are important to the pathogenesis of IE, such as a fully functional immune system and clotting cascade as well as flow conditions within the heart, are difficult to replicate *in vitro* and thus, in many cases findings should be confirmed using *in vivo* IE models. IE has been induced in mice (Liesenborghs et al., 2019; Schwarz et al., 2021), rats (Heraief et al., 1982; Xiong et al., 2005; Veloso et al., 2011; Augustin et al., 2013), rabbits (Durack et al., 1973; Crosby et al., 2016), pigs (Johnson et al., 1986; Christiansen et al., 2013), dogs (Highman et al., 1956), horses (Else and Holmes, 1972) and opossums (Vakilzadeh et al., 1970). *In vivo* IE models have been extensively used for wide-ranging research questions from pathogenesis to treatment outcomes (Lerche et al., 2021). To induce NVE, a catheter-based approach is almost exclusively used to initially cause mechanical damage on aortic valves to create a non-bacterial thrombus and then subsequently bacteria are introduced intravenously (Augustin et al., 2013). In one murine NVE model that sought to mimic valvular inflammation-induced infection, the catheter was used to infuse histamine at the aortic valve site for 5 min while sustaining mechanical injury only during that time window (Liesenborghs et al., 2019). A controversial aspect of the catheter-based approach is that in many studies the catheter remained in place for the entirety of the experiments, i.e., from infection progression to sacrifice, and raises criticism on whether this more represents PVE or CDRIE than NVE (Sande, 1999; Schwarz et al., 2020). To address this, one study modified the catheter approach in four aspects to assess the influence of valvular damage and foreign material presence in mice, and although differences in infection were found, whether either approach best represents NVE or PVE was not concluded (Schwarz et al., 2020).

Another controversial aspect is that a large majority of IE *in vivo* studies used laboratory strains to induce infection. Laboratory-derived strains have been shown to possess genetic changes leading to phenotypic variation among entire strain pedigrees and loss of original and typical *in vivo* virulence (Baek et al., 2013). Furthermore, many of these commonly used laboratory strains were not derived from or known to be IE infections, for example *S. aureus Newman* isolated in 1952 from a human infection type unrecorded or *S. aureus 6850* isolated during a human infection that progressed from skin abscess to osteomyelitis and sepsis. Schwarz et al. set out to research similarities between *in vitro* and *in vivo* behavior of two *S. aureus* strains (Schwarz et al., 2020). Conversely, the isolate with non-aggressive characteristics *in vitro* demonstrated to be the most “aggressive” by fast tissue destruction and immune cell infiltration *in vivo*, compared to the other isolate. It was concluded that the *in vitro* situation cannot directly be translated to the *in vivo* situation, and pathogenic interaction and immune response pathways should be evaluated in future research. However, it would be of interest if *in vivo* studies were repeated with *S. aureus* directly isolated from patients with recent definite IE or animal specific-derived IE strains (Trube et al., 2019) instead of laboratory strains.

Limitations of *in vivo* models do exist because ultimately animals are not a complete human representative. Pigs and rabbits are thought to have a close resembling cardiovascular and immune system to humans (Esteves et al., 2018; Pabst, 2020). The human immune system matches that of the pig by more than 80%, where this is only 10% in mice (Dawson, 2011) and further it is known that mice respond differently to bacterial toxins (Salgado-Pabón and Schlievert, 2014). Yet, mice and rats have been used extensively in IE research, most likely because rodents are less expensive and easier to genetically modify. Differences between animal and human should always be taken into consideration when interpreting *in vivo* IE animal model results. Another consideration is that IE in humans is often accompanied by co-morbidities, such as diabetes mellitus. Hanses et al. used a diabetic rat model to characterize *S. aureus* endocardial biofilms and showed that IE was more severe in diabetic rats compared to non-diabetic rats (Hanses et al., 2014). The impact of other co-morbidities should also be further investigated considering that the increasing prevalence of IE is associated with the increasing elderly population that inherently have more co-morbidities that predispose them to IE.

## Future directions

Several promising new strategies to increase diagnostic and therapeutic potential are under development. It must be noted that though many seem promising, most still require further verification for IE in human clinical trials. There is clinical trial support for switching from intravenous to combinational oral antibiotic therapy using two antibiotics with different mechanisms of action in stable left-sided IE patients (Bundgaard et al., 2019; Iversen et al., 2019). For mono- or combination therapy using approved new-generation (last 10 years) antibiotics against gram-positive bacteria, limited IE-focused clinical data exists or larger prospective trials are needed and thus, should only be considered if resistance, allergy, or clinical/microbiological failure is present (Bloem et al., 2021).

For diagnosis, a novel probe for PET/CT imaging that is specific for bacteria has shown preclinical promise in a *S. aureus*-induced aortic valve endocarditis CD-1 mouse model (Wardak et al., 2020). This 6′′-[18F]Fluoromaltotriose probe, targeting the maltodextrin transporter, showed a 2.3 fold increase in tracer uptake in the aortic valve compared with the non-IE control group. After 20 days of antibiotic treatment with vancomycin, the probe signal returned to baseline values. The use of this probe could potentially be used as a diagnostic tool and for treatment monitoring in patients with IE.

Dabigatran, a thrombin inhibitor, has been extensively studied in preclinical IE *in vivo* studies with promising results (Lerche et al., 2021). Panizzi et al. was able to develop a targeted imaging agent based on this thrombin inhibitor (Panizzi et al., 2020). The radioisotope fluorine-18 was coupled to dabigatran to produce a PET imaging agent, or a fluorochrome was attached to synthesize a near-infrared imaging agent for intravital microscopy. With these new imaging methods, monoclonal antibodies raised against the virulence factors staphylocoagulase and von Willebrand factor-binding protein were shown to inhibit the conversion of fibrinogen into fibrin *in vivo* murine and porcine IE models (Panizzi et al., 2020). Direct thrombin inhibitors have been demonstrated to be safe in patients with *S. aureus* bacteremia (Peetermans et al., 2018) and lower the incidence of developing *S. aureus* bacteremia in patients with atrial fibrillation on anticoagulants (Butt et al., 2021). These results are all encouraging, yet their efficacy and potential role, whether prophylactic and/or therapeutic, need to be clinically determined specific for NVE, PVE, and CDRIE.

Another potential theranostic avenue for IE, called sonobactericide (Lattwein et al., 2020), is focused on the removal of IE biofilms, non-invasively, using ultrasound-activated lipid-coated microbubbles (1–10 µm in diameter), clinically approved as ultrasound contrast agents. Non-targeted microbubbles exposed to focused and unfocused ultrasound have been shown to be effective against *in vitro* biofilms produced from clinical IE *S. aureus* isolates (Lattwein et al., 2022). Ultrasound-induced microbubble displacement resulted in up to 84% biofilm degradation (Lattwein et al., 2022), whereas the infected clot *in vitro* IE model mentioned previously in section 6.5 required the addition of thrombolytics to sonobactericide with an efficacy up to 97% (Lattwein et al., 2018). Thrombolytics currently remain contraindicated for IE, however support exists for its use as a therapeutic agent (Lerche et al., 2021). Vancomycin-decorated microbubbles are also developed to specifically bind to the cell wall of gram-positive bacteria (Kouijzer et al., 2021). Proof-of-concept was demonstrated using a patient-derived IE. *S. aureus* isolate to culture biofilms under flow conditions (5 dyn/cm^2^). Theranostic potential was firstly demonstrated by the significant increase in signal detection with vancomycin-decorated microbubbles using a high-frequency pre-clinical ultrasound scanner. Secondly, very few targeted microbubbles (approximately 1–4) exposed to ultrasound under flow conditions were necessary to achieve a biofilm reduction of up to 28%. It is possible that a higher concentration could result in higher amount of reduction.

Anti-platelet therapy has promise as a potential adjuvant therapy in IE prevention. This therapy inhibits bacteria-platelet interactions and can thereby inhibit biofilm growth. The anti-platelet drug aspirin via its metabolite salicylic acid suppresses *S. aureus* virulence genes, which has been shown to interfer with biofilm formation in a rabbit model (Kupferwasser et al., 1999). However, this effect was not observed in a similar study performed in rats without using another anti-platelet drug, ticlopidine (Veloso et al., 2015). Clinical studies using aspirin in patients with a high IE risk remain to have contradicting results as clearly summarized in a mini review by Leeten et al. (Leeten et al., 2021). Another anti-platelet drug, ticagrelor, a reversible platelet adenosine diphosphate P2Y_12_ receptor inhibitor, has shown bactericidal activity against multiple gram positive bacteria *in vitro*, including MSSA and MRSA (Lancellotti et al., 2019). The same study observed biofilm growth inhibition in mice with implants pre-infected with *S. aureus*. Further investigations are needed to determine if anti-platelet therapy is beneficial in the prevention and treatment of IE.

Another promising therapy, which in the future might be used as an adjuvant therapy for IE, is bacteriophage therapy (Plumet et al., 2022). A first-in-patient case study describes a LVAD patient with a persistent open-chest *S. aureus* device infection (Aslam et al., 2019). The patient was successfully treated with AB-SA01 bacteriophages intravenously without any adverse effects. The safety of this AB-SA01 bacteriophage therapy was further examined in a single-arm non-comparative trial including 13 patients diagnosed with *S. aureus* bacteremia, six were also diagnosed with IE (four PVE patients) (Petrovic Fabijan et al., 2020). Eight of the included patients showed clinical improvement and in eleven patients inflammatory markers declined. Although these results show promise, it remains challenging to evaluate the precise effect of this adjuvant therapy since patients are simultaneously under high-concentration antibiotic therapy.

Recently, a bacteriophage-derived lysin was tested for safety and efficacy in a patient cohort with *S. aureus* blood stream infections, including patients with left- and right-sided IE. The tested lysin (cell wall hydrolase) called exebacase, is the first non-antibiotic antimicrobial direct lytic agent tested in a randomized, double-blind, placebo-controlled proof-of-concept study (Fowler et al., 2020). In the blood stream infections and right-sided endocarditis patient subgroup 80% were found to be clinical responders to the exebacase adjuvant therapy, which was 20.5% higher compared to antibiotics alone (59.5%) (Fowler et al., 2020). After 2 weeks, the clinical responder rate for methicillin-resistant *S. aureus* (MRSA) subgroup receiving exebacase and standard-of-care antibiotics was 42.8% higher compared to antibiotics alone. For the methicillin-sensitive *S. aureus* (MSSA) subgroups, the enhanced treatment effect with the addition of exebacase to the antibiotic regime was not observed. Conversely, an *in vitro* study comparing five MRSA and five MSSA isolates treated with antibiotics combined with exebacase observed synergistic enhancements for both MRSA and MSSA treatment groups (Watson et al., 2020). This underscores the importance of pathogenic interaction and immune response pathways in the (human) *in vivo* setting. In a study using an experimental rabbit MRSA IE model, four of the six animals treated with exebacase and daptomycin showed vegetation size reduction or stabilization (Shah S. U. et al., 2020). A similar rabbit MRSA IE model study using daptomycin and a different lysin, LSVT-1701, also found similar promising results (Huang David et al., 2021). Further studies should be done using multiple strains of MRSA as well as MSSA to better understand its potential for effective clinical application. Exebacase and other bacteriophage-derived products could potentially be a new adjuvant therapy for patients with MRSA IE, however further focused confirmation clinical studies are needed. Other possible future directions for IE treatment include, but not limited to, shockwave therapy (Gnanadhas et al., 2015; Qi et al., 2016), and hyperbaric oxygen therapy (Lerche et al., 2017).

## Conclusion

Infective endocarditis (IE) is a life-threatening microbial infection of native and prosthetic heart valves, endocardial surface, and/or indwelling cardiac devices. Prevalence of IE is increasing and mortality remains high despite technological advances. Often IE literature focuses on native valve IE and/or prosthetic valve IE, cardiac device-related IE, or can be undefined. This review provides an updated overview of all three IE stratifications together and separately concerning clinical presentation, diagnosis, causative pathogens, treatment, and outcomes. Having all three stratifications in one review allows for clear visualization on the similarities and dis-similarities between each, which was found in all clinical domains and highlights the continued importance of stratifying based on infected material. Another strength of this review is that only recent literature was used, almost exclusively within the last 5 years, which the contents and discussion within then reflect current trends in IE infection management. Further, current and novel investigative developments and innovative strategies showing promise to improve diagnostic pipeline and therapeutic outcomes are discussed. Overall, the findings presented in this review provides an overview that will potentiate discussion on IE relating to the different infected materials, ultimately to help towards deriving better diagnostic strategies and treatment management.

## References

[B1] Abdelghani M. Nassif M. Blom N. A. Van Mourik M. S. Straver B. Koolbergen D. R. (2018). Infective endocarditis after Melody valve implantation in the pulmonary position: A systematic review. J. Am. Heart Assoc. 7. 10.1161/JAHA.117.008163 PMC6064882 29934419

[B2] Abdelhady W. Bayer A. S. Seidl K. Nast C. C. Kiedrowski M. R. Horswill A. R. (2013). Reduced vancomycin susceptibility in an in vitro catheter-related biofilm model correlates with poor therapeutic outcomes in experimental endocarditis due to methicillin-resistant *Staphylococcus aureus* . Antimicrob. Agents Chemother. 57, 1447–1454. 10.1128/aac.02073-12 23295925 PMC3591927

[B3] Abegaz T. M. Bhagavathula A. S. Gebreyohannes E. A. Mekonnen A. B. Abebe T. B. (2017). Short- and long-term outcomes in infective endocarditis patients: A systematic review and meta-analysis. BMC Cardiovasc Disord. 17, 1–12. 10.1186/s12872-017-0729-5 29233094 PMC5728061

[B4] Abikhzer G. Martineau P. Grégoire J. Finnerty V. Harel F. Pelletier-Galarneau M. (2020). [18F]FDG-PET CT for the evaluation of native valve endocarditis. J. Nucl. Cardiol. 10.1007/s12350-020-02092-6 32180137

[B5] Aguilera J. Hutt E. Jaber W. A. (2021). Imaging of cardiac device-related infection. Front. Cardiovasc Med. 8, 729786. 10.3389/fcvm.2021.729786 34504881 PMC8421771

[B6] Ahtela E. Oksi J. Sipilä J. Rautava P. Kytö V. (2019). Occurrence of fatal infective endocarditis: A population-based study in Finland. BMC Infect. Dis. 19, 987. 10.1186/s12879-019-4620-0 31752727 PMC6873758

[B7] Akin S. Muslem R. Constantinescu A. A. Manintveld O. C. Birim O. Brugts J. J. (2018). 18F-FDG PET/CT in the diagnosis and management of continuous flow left ventricular assist device infections: A case Series and review of the literature. ASAIO J. 64, e11–e19. 10.1097/mat.0000000000000552 28234643 PMC5839716

[B8] Ali S. George L. K. Das P. Koshy S. K. (2011). Intracardiac echocardiography: Clinical utility and application. Echocardiography 28, 582–590. 10.1111/j.1540-8175.2011.01395.x 21564275

[B9] Almatrafi M. A. Alsahaf N. Alsharif E. J. Sayed J. A. Telmesani A. M. A. Alidrisi D. (2021). Adjunctive rifampin therapy for native valve *Staphylococcus aureus* endocarditis in a neonate: A case report and literature review. Clin. Case Rep. 9, e04902. 10.1002/ccr3.4902 34631085 PMC8489504

[B10] Ammannaya G. K. K. Sripad N. (2019). Fungal endocarditis: what do we know in 2019? 77. Warsaw: Kardiologia Polska. 10.33963/kp.14869 31215523

[B11] Armstrong C. Kuhn T. C. Dufner M. Ehlermann P. Zimmermann S. Lichtenstern C. (2021). The diagnostic benefit of 16S rDNA PCR examination of infective endocarditis heart valves: A cohort study of 146 surgical cases confirmed by histopathology. Clin. Res. Cardiol. 110, 332–342. 10.1007/s00392-020-01678-x 32488586 PMC7906935

[B12] Aslam S. Pretorius V. Lehman S. M. Morales S. Schooley R. T. (2019). Novel bacteriophage therapy for treatment of left ventricular assist device infection. J. Heart Lung Transplant. 38, 475–476. 10.1016/j.healun.2019.01.001 30661974

[B13] Augustin P. Alsalih G. Launey Y. Delbosc S. Louedec L. Ollivier V. (2013). Predominant role of host proteases in myocardial damage associated with infectious endocarditis induced by *Enterococcus faecalis* in a rat model. Infect. Immun. 81, 1721–1729. 10.1128/iai.00775-12 23478315 PMC3647991

[B14] Babeș E. E. Lucuța D. A. Petcheși C. D. ZAHA A. A. Ilyes C. Jurca A. D. (2021). Clinical Features and Outcome of Infective Endocarditis in a University Hospital in Romania, 57. Medicina. 10.3390/medicina57020158 PMC7916483 33578787

[B15] Baddour L. M. Wilson W. R. Bayer A. S. Fowler V. G., JR. Tleyjeh I. M. Rybak M. J. (2015). Infective endocarditis in adults: Diagnosis, antimicrobial therapy, and management of complications: A Scientific statement for Healthcare Professionals from the American heart association. Circulation 132, 1435–1486. 10.1161/cir.0000000000000296 26373316

[B16] Bæk K. T. Frees D. Renzoni A. Barras C. Rodriguez N. Manzano C. (2013). Genetic variation in the *Staphylococcus aureus* 8325 strain lineage revealed by whole-genome sequencing. PLoS One 8, e77122. 10.1371/journal.pone.0077122 24098817 PMC3786944

[B17] Bin Abdulhak A. A. Qazi A. H. Tleyjeh I. M. (2018). Current Treatment Options in Cardiovascular Medicine, 20. 10.1007/s11936-018-0668-1 Workup and management of native and prosthetic valve endocarditis 30083823

[B18] Bloem A. Bax H. I. Yusuf E. Verkaik N. J. (2021). New-generation antibiotics for treatment of gram-positive infections: A review with focus on endocarditis and osteomyelitis. J. Clin. Med. 10. 10.3390/jcm10081743 PMC8074169 33920526

[B19] Blomström-Lundqvist C. Traykov V. Erba P. A. Burri H. Nielsen J. C. Bongiorni M. G. (2020). European heart Rhythm association (EHRA) international consensus document on how to prevent, diagnose, and treat cardiac implantable electronic device infections-endorsed by the heart Rhythm Society (HRS), the Asia Pacific heart Rhythm Society (APHRS), the Latin American heart Rhythm Society (LAHRS), international Society for cardiovascular infectious diseases (ISCVID) and the European Society of clinical microbiology and infectious diseases (ESCMID) in collaboration with the European association for Cardio-thoracic surgery (EACTS). Eur. J. Cardiothorac. Surg. 57, e1. 10.1093/ejcts/ezz296 31724720

[B20] Blomström-Lundqvist C. Traykov V. Erba P. A. Burri H. Nielsen J. C. Bongiorni M. G. (2020). European heart Rhythm association (EHRA) international consensus document on how to prevent, diagnose, and treat cardiac implantable electronic device infections-endorsed by the heart Rhythm Society (HRS), the Asia Pacific heart Rhythm Society (APHRS), the Latin American heart Rhythm Society (LAHRS), international Society for cardiovascular infectious diseases (ISCVID), and the European Society of clinical microbiology and infectious diseases (ESCMID) in collaboration with the European association for Cardio-thoracic surgery (EACTS). Eur. Heart J. 41, 2012–2032. 10.1093/eurheartj/ehaa010 32101604

[B21] Bohbot Y. Peugnet F. Lieu A. Carbone A. Mouhat B. Philip M. (2021). Characteristics and prognosis of patients with left-sided native Bivalvular infective endocarditis. Can. J. Cardiol. 37, 292–299. 10.1016/j.cjca.2020.03.046 32835685

[B22] Boljevic D. Barac A. Vukovic P. Kojic D. Bojic M. Micic J. (2019). A rare case of pacemaker lead endocarditis successfully treated with open heart surgery. J. Infect. Dev. Ctries. 13, 1068–1071. 10.3855/jidc.11941 32087081

[B23] Bouajila S. Chalard A. Dauphin C. (2017). Usefulness of intracardiac echocardiography for the diagnosis of infective endocarditis following percutaneous pulmonary valve replacement. Cardiol. Young 27, 1406–1409. 10.1017/s1047951117000403 28322179

[B24] Boujelben I. Gdoura R. Hammami A. (2018). A broad-range PCR technique for the diagnosis of infective endocarditis. Braz. J. Microbiol. 49, 534–543. 10.1016/j.bjm.2017.03.019 29429763 PMC6111453

[B25] Breitkopf C. Hammel D. Scheld H. H. Peters G. Becker K. (2005). Impact of a molecular approach to improve the microbiological diagnosis of infective heart valve endocarditis. Circulation 111, 1415–1421. 10.1161/01.cir.0000158481.07569.8d 15753218

[B26] Brinkman C. L. Vergidis P. Uhl J. R. Pritt B. S. Cockerill F. R. Steckelberg J. M. (2013). PCR-electrospray ionization mass spectrometry for direct detection of pathogens and antimicrobial resistance from heart valves in patients with infective endocarditis. J. Clin. Microbiol. 51, 2040–2046. 10.1128/jcm.00304-13 23596241 PMC3697732

[B27] Brouqui P. Raoult D. (2001). Endocarditis due to rare and fastidious bacteria. Clin. Microbiol. Rev. 14, 177–207. 10.1128/cmr.14.1.177-207.2001 11148009 PMC88969

[B28] Bruns V. Franz D. Kuritcyn P. Wiesmann V. Rathke M. Wittenberg T. (2021). Towards computer aided diagnosis of infective endocarditis in whole-slide images of heart valve tissue using FISH. Curr. Dir. Biomed. Eng. 7, 468–471. 10.1515/cdbme-2021-2119

[B29] Bundgaard H. Ihlemann N. Gill S. U. Bruun N. E. Elming H. Madsen T. (2019). Long-term outcomes of partial oral treatment of endocarditis. N. Engl. J. Med. 380, 1373–1374. 10.1056/nejmc1902096 30883059

[B30] Butt J. H. Fosbøl E. L. Verhamme P. Gerds T. A. Iversen K. Bundgaard H. (2021). Dabigatran and the risk of *Staphylococcus aureus* bacteremia: A nationwide cohort study. Clin. Infect. Dis. 73, 480–486. 10.1093/cid/ciaa661 32478836

[B31] Butt J. H. Ihlemann N. De Backer O. Søndergaard L. Havers-Borgersen E. Gislason G. H. (2019). Long-Term risk of infective endocarditis after transcatheter aortic valve replacement. J. Am. Coll. Cardiol. 73, 1646–1655. 10.1016/j.jacc.2018.12.078 30947917

[B32] Cahill T. J. Baddour L. M. Habib G. Hoen B. Salaun E. Pettersson G. B. (2017). Challenges in infective endocarditis. J. Am. Coll. Cardiol. 69, 325–344. 10.1016/j.jacc.2016.10.066 28104075

[B33] Cahill T. J. Prendergast B. D. (2016). Infective endocarditis. Lancet 387, 882–893. 10.1016/s0140-6736(15)00067-7 26341945

[B34] Cahill T. J. Raby J. Jewell P. D. Brennan P. F. Banning A. P. Byrne J. (2022). Risk of infective endocarditis after surgical and transcatheter aortic valve replacement. Heart 108, 639–647. 10.1136/heartjnl-2021-320080 35058295

[B35] Cai S. Yang Y. Pan J. Miao Q. Jin W. Ma Y. (2021). The clinical value of valve metagenomic next-generation sequencing when applied to the etiological diagnosis of infective endocarditis. Ann. Transl. Med. 9, 1490. 10.21037/atm-21-2488 34805352 PMC8573444

[B36] Calais J. Touati A. Grall N. Laouénan C. Benali K. Mahida B. (2019). Diagnostic impact of 18F-fluorodeoxyglucose positron emission tomography/computed tomography and white blood cell SPECT/computed tomography in patients with suspected cardiac implantable electronic device chronic infection. Circ. Cardiovasc Imaging 12, e007188. 10.1161/CIRCIMAGING.117.007188 31291779

[B37] Chambers H. F. Bayer A. S. (2020). Native-valve infective endocarditis. N. Engl. J. Med. 383, 567–576. 10.1056/NEJMcp2000400 32757525

[B38] Chang D. Gabriels J. Laighold S. Williamson A. K. Ismail H. Epstein L. M. (2019). A novel diagnostic approach to a mass on a device lead. Hear. Case Rep. 5, 306–309. 10.1016/j.hrcr.2019.03.001 PMC6587050 31285986

[B39] Cheng J. Hu H. Kang Y. Chen W. Fang W. Wang K. (2018). Identification of pathogens in culture-negative infective endocarditis cases by metagenomic analysis. Ann. Clin. Microbiol. Antimicrob. 17, 43. 10.1186/s12941-018-0294-5 30567558 PMC6300891

[B40] Christiansen J. G. Jensen H. E. Johansen L. K. Kochl J. Koch J. Aalbaek B. (2013). Porcine models of non-bacterial thrombotic endocarditis (NBTE) and infective endocarditis (IE) caused by *Staphylococcus aureus*: A preliminary study. J. Heart Valve Dis. 22, 368–376. 24151763

[B41] Chuang-Smith O. N. Wells C. L. Henry-Stanley M. J. Dunny G. M. (2010). Acceleration of *Enterococcus faecalis* biofilm formation by aggregation substance expression in an *ex vivo* model of cardiac valve colonization. PLoS One 5, e15798. 10.1371/journal.pone.0015798 21209892 PMC3012704

[B42] Claes J. Vanassche T. Peetermans M. Liesenborghs L. Vandenbriele C. Vanhoorelbeke K. (2014). Adhesion of *Staphylococcus aureus* to the vessel wall under flow is mediated by von Willebrand factor-binding protein. Blood 124, 1669–1676. 10.1182/blood-2014-02-558890 24951431 PMC7025350

[B43] Coenye T. Nelis H. J. (2010). In vitro and in vivo model systems to study microbial biofilm formation. J. Microbiol. Methods 83, 89–105. 10.1016/j.mimet.2010.08.018 20816706

[B44] Crosby H. A. Schlievert P. M. Merriman J. A. King J. M. Salgado-Pabón W. Horswill A. R. (2016). The *Staphylococcus aureus* Global Regulator MgrA Modulates Clumping and virulence by controlling surface protein expression. PLoS Pathog. 12, e1005604. 10.1371/journal.ppat.1005604 27144398 PMC4856396

[B45] Cuervo G. Escrihuela-Vidal F. Gudiol C. Carratalà J. (2021). Current challenges in the management of infective endocarditis. Front. Med. 8. 10.3389/fmed.2021.641243 PMC7937698 33693021

[B46] D'humieres C. Salmona M. Delliere S. Leo S. Rodriguez C. Angebault C. (2021). The potential role of clinical metagenomics in infectious diseases: Therapeutic Perspectives. Drugs 81, 1453–1466. 34328626 10.1007/s40265-021-01572-4 PMC8323086

[B47] Das A. S. McKeown M. Jordan S. A. Li K. Regenhardt R. W. Feske S. K. (2022). Risk factors for neurological complications in left-sided infective endocarditis. J. Neurological Sci. 442, 120386. 10.1016/j.jns.2022.120386 36030704

[B48] Dawson H. (2011). The minipig in biomedical research. Boca Raton: CRC Press.

[B49] De Camargo R. A. Sommer Bitencourt M. Meneghetti J. C. Soares J. Gonçalves L. F. T. Buchpiguel C. A. (2019). The role of 18F-fluorodeoxyglucose positron emission tomography/computed tomography in the diagnosis of left-sided endocarditis: Native vs prosthetic valves endocarditis. Arlington: Clinical Infectious Diseases. 10.1093/cid/ciz267 30949690

[B50] Defauw R. J. Tomšič A. van Brakel T. J. Marsan N. A. Klautz R. J. M. Palmen M. (2020). A structured approach to native mitral valve infective endocarditis: Is repair better than replacement? Eur. J. Cardiothorac. Surg. 58, 544–550. 10.1093/ejcts/ezaa079 32333009 PMC7453034

[B51] Dell'Aquila A. M. Mastrobuoni S. Alles S. Wenning C. Henryk W. Schneider S. R. B. (2016). Contributory role of fluorine 18-fluorodeoxyglucose positron emission tomography/computed tomography in the diagnosis and clinical management of infections in patients supported with a continuous-flow left ventricular assist device. Ann. Thorac. Surg. 101, 87–94. 10.1016/j.athoracsur.2015.06.066 26433521

[B52] Desimone D. C. Lahr B. D. Anavekar N. S. Sohail M. R. Tleyjeh I. M. Wilson W. R. (2021). Temporal Trends of Infective Endocarditis in Olmsted County, Minnesota, Between 1970 and 2018: A Population-Based Analysis, 8. Arlington: Open Forum Infectious Diseases. 10.1093/ofid/ofab038 PMC7944350 33728357

[B53] DeSimone D. C. Sohail M. R. (2018). Approach to diagnosis of cardiovascular implantable-electronic-device infection. J. Clin. Microbiol. 56. 10.1128/JCM.01683-17 PMC6018346 29695526

[B54] DeSimone D. C. Sohail M. R. Mulpuru S. K. (2019). Contemporary management of cardiac implantable electronic device infection. Heart 105, 961–965. 10.1136/heartjnl-2017-312146 30755468

[B55] Di Domenico E. G. Rimoldi S. G. Cavallo I. D’Agosto G. Trento E. Cagnoni G. (2019). Microbial biofilm correlates with an increased antibiotic tolerance and poor therapeutic outcome in infective endocarditis. BMC Microbiol. 19. 10.1186/s12866-019-1596-2 PMC6802308 31638894

[B56] Döring M. Richter S. Hindricks G. (2018). The diagnosis and treatment of pacemaker-associated infection. Dtsch. Arztebl Int. 115, 445–452. 10.3238/arztebl.2018.0445 30017027 PMC6071306

[B57] Durack D. T. Beeson P. B. Petersdorf R. G. (1973). Experimental bacterial endocarditis. 3. Production and progress of the disease in rabbits. Br. J. Exp. Pathol. 54, 142–151. 4700697 PMC2072580

[B58] Duval X. le Moing V. Tubiana S. Esposito-Farèse M. Ilic-Habensus E. Leclercq F. (2021). Impact of Systematic Whole-body 18F-Fluorodeoxyglucose PET/CT on the Management of Patients Suspected of Infective Endocarditis: The Prospective Multicenter TEPvENDO Study, 73. Arlington: Clinical Infectious Diseases. 10.1093/cid/ciaa666 32488236

[B59] Eberle F. C. Kanyildiz M. Schnabl S. M. Schulz C. Häfner H. M. Adam P. (2014). Dreidimensionale (3D) Histologie im Routineverfahren: praktische Durchführung und deren Evaluation. JDDG J. der Deutschen Dermatologischen Gesellschaft 12, 1028–1036. 10.1111/ddg.12466_suppl 25354011

[B60] Eichenberger E. M. Degner N. Scott E. R. Ruffin F. Franzone J. Sharma-Kuinkel B. (2022). Microbial cell-free DNA identifies the causative pathogen in infective endocarditis and remains detectable longer than Conventional blood culture in patients with prior antibiotic therapy. Clin. Infect. Dis. 10.1093/cid/ciac426 PMC10169441 35684984

[B61] El Gabry M. Haidari Z. Mourad F. Nowak J. Tsagakis K. Thielmann M. (2019). Outcomes of mitral valve repair in acute native mitral valve infective endocarditis. Interact. Cardiovasc. Thorac. Surg. 29. 10.1093/icvts/ivz187 31369076

[B62] El Kadi S. van den Buijs D. M. F. Meijers T. Gilbers M. D. Bekkers S. C. A. M. van Melle J. P. (2020). Infective endocarditis in The Netherlands: Current epidemiological profile and mortality : An analysis based on partial ESC EORP collected data. Neth Heart J. 28, 526–536. 10.1007/s12471-020-01431-z 32504340 PMC7494701

[B63] Ellis M. E. Al-Abdely H. Sandridge A. Greer W. Ventura W. (2001). Fungal endocarditis: Evidence in the world literature, 1965-1995. Clin. Infect. Dis. 32, 50–62. 10.1086/317550 11118386

[B64] Else R. W. Holmes J. R. (1972). Cardiac pathology in the horse. Equine Vet. J. 4, 1–8. 10.1111/j.2042-3306.1972.tb03868.x 4650883

[B65] Ely D. Tan C. D. Rodriguez E. R. Hussain S. Pettersson G. Gordon S. (2016). Histological Findings in Infective Endocarditis, 3. Arlington: Open Forum Infectious Diseases. 10.1093/ofid/ofw172.814

[B66] Erba P. A. Slart R. H. J. A. (2020). Radiolabeled-white blood cell imaging in cardiac device-related Infective Endocarditis: Worth all the Effort? JACC Cardiovasc Imaging 13, 1752–1754. 10.1016/j.jcmg.2020.02.033 32563659

[B67] Erbel R. Rohmann S. Drexler M. Mohr-Kahaly S. Gerharz C. D. Iversen S. (1988). Improved diagnostic value of echocardiography in patients with infective endocarditis by transoesophageal approach. A prospective study. Eur. Heart J. 9, 43–53. 10.1093/ehj/9.1.43 3345769

[B68] Esteves P. J. Abrantes J. Baldauf H. M. BenMohamed L. Chen Y. Christensen N. (2018). The wide utility of rabbits as models of human diseases. Exp. Mol. Med. 50, 1–10. 10.1038/s12276-018-0094-1 PMC5964082 29789565

[B69] Fagman E. van Essen M. Fredén Lindqvist J. Snygg-Martin U. Bech-Hanssen O. Svensson G. (2016). 18F-FDG PET/CT in the diagnosis of prosthetic valve endocarditis. Int. J. Cardiovasc Imaging 32, 679–686. 10.1007/s10554-015-0814-8 26611107

[B70] Faraji R. Behjati‐Ardakani M. Moshtaghioun S. M. Kalantar S. M. Namayandeh S. M. Soltani M. (2018). The diagnosis of microorganism involved in infective endocarditis (IE) by polymerase chain reaction (PCR) and real‐time PCR: A systematic review. Kaohsiung J Med Scie 34, 71–78. 10.1016/j.kjms.2017.09.011 PMC11915617 29413230

[B71] Fauchier L. Bisson A. Herbert J. Lacour T. Bourguignon T. Etienne C. S. (2020). Incidence and outcomes of infective endocarditis after transcatheter aortic valve implantation versus surgical aortic valve replacement. Clin. Microbiol. Infect. 26, 1368–1374. 10.1016/j.cmi.2020.01.036 32036047

[B72] Forestier E. Fraisse T. Roubaud-Baudron C. Selton-Suty C. Pagani L. (2016). Managing infective endocarditis in the elderly: New issues for an old disease. Clin. Interv. Aging 11, 1199–1206. 10.2147/CIA.S101902 27621607 PMC5015881

[B73] Fowler V. G., JR. Das A. F. Lipka-Diamond J. Schuch R. Pomerantz R. Jáuregui-Peredo L. (2020). Exebacase for patients with *Staphylococcus aureus* bloodstream infection and endocarditis. J. Clin. Investig. 130, 3750–3760. 10.1172/jci136577 32271718 PMC7324170

[B74] Galar A. Weil A. A. Dudzinski D. M. Munoz P. Siedner M. J. (2019a). Methicillin-Resistant *Staphylococcus aureus* Prosthetic Valve Endocarditis: Pathophysiology, Epidemiology, Clinical Presentation, Diagnosis, and Management, 32. Washington: Clin Microbiol Rev. 10.1128/cmr.00041-18 PMC6431130 30760474

[B75] Galar A. Weil A. A. Dudzinski D. M. Muñoz P. Siedner M. J. (2019b). Methicillin-resistant *Staphylococcus aureus* prosthetic valve endocarditis: Pathophysiology, epidemiology, clinical presentation, diagnosis, and management. Clin. Microbiol. Rev. 32. 10.1128/CMR.00041-18 PMC6431130 30760474

[B76] Galea N. Bandera F. Lauri C. Autore C. Laghi A. Erba P. A. (2020). Multimodality imaging in the diagnostic work-up of endocarditis and cardiac implantable electronic device (CIED) infection. J. Clin. Med. 9. 10.3390/jcm9072237 PMC7408824 32674517

[B77] Gauduchon V. Chalabreysse L. Etienne J. Célard M. Benito Y. Lepidi H. (2003). Molecular diagnosis of infective endocarditis by PCR amplification and direct sequencing of DNA from valve tissue. J. Clin. Microbiol. 41, 763–766. 10.1128/jcm.41.2.763-766.2003 12574279 PMC149702

[B78] Georgiev S. Ewert P. Eicken A. Hager A. Hörer J. Cleuziou J. (2020). Munich comparative study: Prospective long-term outcome of the transcatheter Melody valve versus surgical pulmonary bioprosthesis with up to 12 Years of Follow-up. Circ. Cardiovasc Interv. 13, e008963. 10.1161/CIRCINTERVENTIONS.119.008963 32600110

[B79] Gnanadhas D. P. Elango M. Janardhanraj S. Srinandan C. S. Datey A. Strugnell R. A. (2015). Successful treatment of biofilm infections using shock waves combined with antibiotic therapy. Sci. Rep. 5, 17440. 10.1038/srep17440 26658706 PMC4674795

[B80] Goenaga Sánchez M. Á. Kortajarena Urkola X. Bouza Santiago E. Muñoz García P. Verde Moreno E. Fariñas Álvarez M. C. (2017). The role of antibiotics. English Edition. Barcelona: Medicina Clínica, 149.Aetiology of renal failure in patients with infective endocarditis 10.1016/j.medcli.2017.03.009 28431897

[B81] Gomes A. Glaudemans A. W. J. M. Touw D. J. van Melle J. P. Willems T. P. Maass A. H. (2017). Diagnostic value of imaging in infective endocarditis: A systematic review. Lancet Infect. Dis. 17, e1. 10.1016/S1473-3099(16)30141-4 27746163

[B82] Gomes A. van Geel P. P. Santing M. Prakken N. H. J. Ruis M. L. van Assen S. (2020). Imaging infective endocarditis: Adherence to a diagnostic flowchart and direct comparison of imaging techniques. J. Nucl. Cardiol. 27, 592–608. 10.1007/s12350-018-1383-8 30066279 PMC7174257

[B83] Granados U. Fuster D. Pericas J. M. Llopis J. L. Ninot S. Quintana E. (2016). Diagnostic accuracy of 18F-FDG PET/CT in infective endocarditis and implantable cardiac electronic device infection: A cross-Sectional study. J. Nucl. Med. 57, 1726–1732. 10.2967/jnumed.116.173690 27261514

[B84] Guenther S. P. W. Cyran C. C. Rominger A. Saam T. Kazmierzcak P. M. Bagaev E. (2015). The relevance of 18F-fluorodeoxyglucose positron emission tomography/computed tomography imaging in diagnosing prosthetic graft infections post cardiac and proximal thoracic aortic surgery. Interact. Cardiovasc Thorac. Surg. 21, 450–458. 10.1093/icvts/ivv178 26174118

[B85] Habib G. Erba P. A. Iung B. Donal E. Cosyns B. Laroche C. (2019). Clinical presentation, aetiology and outcome of infective endocarditis. Results of the ESC-EORP EURO-ENDO (European infective endocarditis) registry: A prospective cohort study. Eur. Heart J. 40, 3222–3232. 10.1093/eurheartj/ehz620 31504413

[B86] Habib G. Hoen B. Tornos P. Thuny F. Prendergast B. Vilacosta I. (2009). Guidelines on the prevention, diagnosis, and treatment of infective endocarditis (new version 2009): The Task force on the prevention, diagnosis, and treatment of infective endocarditis of the European Society of cardiology (ESC). Endorsed by the European Society of clinical microbiology and infectious diseases (ESCMID) and the international Society of Chemotherapy (ISC) for infection and Cancer. Eur. Heart J. 30, 2369–2413. 10.1093/eurheartj/ehp285 19713420

[B87] Habib G. Lancellotti P. Antunes M. J. Bongiorni M. G. Casalta J.-P. Del Zotti F. (2015). 2015 ESC guidelines for the management of infective endocarditis: The Task force for the management of infective endocarditis of the European Society of cardiology (ESC). Endorsed by: European association for Cardio-thoracic surgery (EACTS), the European association of nuclear medicine (EANM). Eur. Heart J. 36, 3075–3128. 10.1093/eurheartj/ehv319 26320109

[B88] Hannachi N. Lepidi H. Fontanini A. Takakura T. Bou-Khalil J. Gouriet F. (2020)., 9. Cells. 10.3390/cells9081899 A novel approach for detecting Unique variations among infectious bacterial species in endocarditic cardiac valve vegetation Cells PMC7464176 32823780

[B89] Hanses F. Roux C. Dunman P. M. Salzberger B. Lee J. C. (2014). *Staphylococcus aureus* gene expression in a rat model of infective endocarditis. Genome Med. 6, 93. 10.1186/preaccept-4819325051343079 25392717 PMC4228149

[B90] Harrison J. J. Stremick C. A. Turner R. J. Allan N. D. Olson M. E. Ceri H. (2010). Microtiter susceptibility testing of microbes growing on peg lids: A miniaturized biofilm model for high-throughput screening. Nat. Protoc. 5, 1236–1254. 10.1038/nprot.2010.71 20595953

[B91] Hascoet S. Mauri L. Claude C. Fournier E. Lourtet J. Riou J. Y. (2017). Infective endocarditis risk after percutaneous pulmonary valve implantation with the Melody and Sapien valves. JACC Cardiovasc. Interv. 10, 510–517. 10.1016/j.jcin.2016.12.012 28279319

[B92] Héraïef E. Glauser M. P. Freedman L. R. (1982). Natural history of aortic valve endocarditis in rats. Infect. Immun. 37, 127–131. 10.1128/iai.37.1.127-131.1982 7049946 PMC347499

[B93] Heriot G. S. Newcomb A. Darby J. Wilson A. Tong S. Y. C. Cheng A. C. (2019). Early transthoracic echocardiography has useful prognostic value in left-sided native valve endocarditis despite limited diagnostic performance. Eur. J. Clin. Microbiol. Infect. Dis. 38, 1569–1575. 10.1007/s10096-019-03589-w 31140069

[B94] Highman B. Roshe J. Altland P. D. (1956). Production of endocarditis with *Staphylococcus aureus* and Streptococcus mitis in dogs with aortic insufficiency. Circulation Res. 4, 250–256. 10.1161/01.res.4.3.250 13317014

[B95] Holcman K. Szot W. Rubiś P. Leśniak-Sobelga A. Hlawaty M. Wiśniowska-Śmiałek S. (2019). 99mTc-HMPAO-labeled leukocyte SPECT/CT and transthoracic echocardiography diagnostic value in infective endocarditis. Int. J. Cardiovasc Imaging 35, 749–758. 10.1007/s10554-018-1487-x 30382475 PMC6482119

[B96] Holland T. L. Baddour L. M. Bayer A. S. Hoen B. Miro J. M. Fowler V. G. (2016). Infective endocarditis. Nat. Rev. Dis. Prim. 2, 16059. 10.1038/nrdp.2016.59 27582414 PMC5240923

[B97] Holler J. G. Pedersen L. K. Calum H. Nielsen J. B. Tvede M. Schønning K. (2011). Using MALDI-TOF mass spectrometry as a rapid and accurate diagnostic tool in infective endocarditis: A case report of a patient with mitral valve infective endocarditis caused by Abiotrophia defectiva. Scand. J. Infect. Dis. 43, 234–237. 10.3109/00365548.2010.535559 21091125

[B98] Horgan S. J. Mediratta A. Gillam L. D. (2020a). Cardiovascular imaging in infective endocarditis: A multimodality approach. Circ. Cardiovasc Imaging 13, e008956. 10.1161/CIRCIMAGING.120.008956 32683888

[B99] Horgan S. J. Mediratta A. Gillam L. D. (2020b). Cardiovascular imaging in infective endocarditis: A multimodality approach. Circ. Cardiovasc Imaging 13, e008956. 10.1161/CIRCIMAGING.120.008956 32683888

[B100] Houpikian P. Raoult D. (2005). Blood culture-negative endocarditis in a reference center. Med. Baltim. 84, 162–173. 10.1097/01.md.0000165658.82869.17 15879906

[B101] Hryniewiecki T. Zatorska K. Abramczuk E. Zakrzewski D. Szymański P. Kuśmierczyk M. (2019). The usefulness of cardiac CT in the diagnosis of perivalvular complications in patients with infective endocarditis. Eur. Radiol. 29, 4368–4376. 10.1007/s00330-018-5965-2 30643945 PMC6611057

[B102] Huang David B. Gaukel E. Kerzee N. Borroto-Esoda K. Lowry S. Xiong Yan Q. (2021). Efficacy of Antistaphylococcal lysin LSVT-1701 in combination with daptomycin in experimental left-sided infective endocarditis due to methicillin-resistant *Staphylococcus aureus* . Antimicrob. Agents Chemother. 65, e00508–e00521. 10.1128/aac.00508-21 PMC8284455 34097491

[B103] Hubers S. A. DeSimone D. C. Gersh B. J. Anavekar N. S. (2020). Infective endocarditis: A contemporary review. Mayo Clin. Proc. 95, 982–997. 10.1016/j.mayocp.2019.12.008 32299668

[B104] Hussein A. A. Baghdy Y. Wazni O. M. Brunner M. P. Kabbach G. Shao M. (2016). Microbiology of cardiac implantable electronic device infections. JACC Clin. Electrophysiol. 2, 498–505. 10.1016/j.jacep.2016.01.019 29759872

[B105] Huuskonen A. Kaarne M. Vento A. Juvonen T. Raivio P. (2021). Outcomes of surgery for extensive infective endocarditis. J. Cardiac Surg. 10.1111/jocs.16005 34547124

[B106] Huuskonen A. Vento A. Raivio P. (2019). Outcome of early vs delayed surgery for infective mitral endocarditis. J. Card. Surg. 34, 700–707. 10.1111/jocs.14137 31269271

[B107] Ibrahim A. M. Siddique M. S. (2021). Subacute bacterial endocarditis prophylaxis. 30422578

[B108] Ivanovic B. Trifunovic D. Matic S. Petrovic J. Sacic D. Tadic M. (2019). Prosthetic valve endocarditis - a trouble or a challenge? J. Cardiol. 73, 126–133. 10.1016/j.jjcc.2018.08.007 30389305

[B109] Iversen K. Ihlemann N. Gill S. U. Madsen T. Elming H. Jensen K. T. (2019). Partial oral versus intravenous antibiotic treatment of endocarditis. N. Engl. J. Med. 380, 415–424. 10.1056/nejmoa1808312 30152252

[B110] Jahanbakhsh S. Singh N. B. Yim J. Kebriaei R. Smith J. R. Lev K. (2020). Impact of daptomycin Dose exposure alone or in combination with β-Lactams or rifampin against vancomycin-resistant enterococci in an in vitro biofilm model. Antimicrob. Agents Chemother. 64. 10.1128/AAC.02074-19 PMC7179592 32094136

[B111] Jainandunsing J. S. Bergman R. Wilkens J. Wang A. Michielon G. Natour E. (2014). Ventriculo-atrial defect after bioprosthetic aortic valve replacement. J. Cardiothorac. Surg. 9, 137. 10.1186/s13019-014-0137-1 PMC4205768 25274005

[B112] Jędrzejczyk-Patej E. Mazurek M. Kowalski O. Sokal A. Liberska A. Szulik M. (2021). Clinical manifestations of device-related infective endocarditis in cardiac resynchronization therapy recipients. Arch. Med. Sci. 17, 638–645. 10.5114/aoms.2018.75893 34025833 PMC8130459

[B113] Johnson C. M. Bahn R. C. Fass D. N. (1986). Experimental porcine infective endocarditis: Description of a clinical model. Vet. Pathol. 23, 780–782. 10.1177/030098588602300620 3811145

[B114] Karchmer A. W. Chu V. H. Otto C. M. (2018). Prosthetic valve endocarditis: Epidemiology, clinical manifestations, and diagnosis. Alphen aan Den Rijn: UpToDate, 21. Accessed.https://www.uptodate.com/contents/prosthetic-valve-endocarditis-epidemiology-clinical-manifestations-and-diagnosis

[B115] Keller K. von Bardeleben R. S. Ostad M. A. Hobohm L. Munzel T. Konstantinides S. (2017). Temporal trends in the prevalence of infective endocarditis in Germany between 2005 and 2014. Am. J. Cardiol. 119, 317–322. 10.1016/j.amjcard.2016.09.035 27816113

[B116] Khalil H. Soufi S. (2022). Prosthetic valve endocarditis. *StatPearls.* Treasure. Island (FL. 33620808

[B117] Kikhney J. Moter A. (2021). “Quality control in diagnostic fluorescence *in situ* hybridization (FISH) in microbiology,” in Fluorescence in-situ hybridization (FISH) for microbial cells: Methods and concepts. Editors AZEVEDO N. F. ALMEIDA C. (New York, NY: Springer US). 10.1007/978-1-0716-1115-9_20 33576998

[B118] Kim D. H. Tate J. Dresen W. F. Papa F. C., JR. Bloch K. C. Kalams S. A. (2014). Cardiac implanted electronic device-related infective endocarditis: Clinical features, management, and outcomes of 80 consecutive patients. Pacing Clin. Electrophysiol. 37, 978–985. 10.1111/pace.12452 25060820

[B119] Kolb M. Lazarevic V. Emonet S. Calmy A. Girard M. Gaïa N. (2019). Next-generation sequencing for the diagnosis of challenging culture-negative endocarditis. Front. Med. 6, 203. 10.3389/fmed.2019.00203 PMC6763761 31616669

[B120] Kolodner D. Q. Shimbo D. Magnano A. R. (2007). Intracardiac echocardiography in the diagnosis of prosthetic valve endocarditis. Heart 93, 1120. 10.1136/hrt.2006.104091 17699175 PMC1955030

[B121] Kolte D. Goldsweig A. Kennedy K. F. Abbott J. D. Gordon P. C. Sellke F. W. (2018). Comparison of incidence, predictors, and outcomes of early infective endocarditis after transcatheter aortic valve implantation versus surgical aortic valve replacement in the United States. Am. J. Cardiol. 122, 2112–2119. 10.1016/j.amjcard.2018.08.054 30292332

[B122] Koneru S. Huang S. S. Oldan J. Betancor J. Popovic Z. B. Rodriguez L. L. (2018). Role of preoperative cardiac CT in the evaluation of infective endocarditis: Comparison with transesophageal echocardiography and surgical findings. Cardiovasc Diagn Ther. 8, 439–449. 10.21037/cdt.2018.07.07 30214859 PMC6129819

[B123] Kouijzer J. J. Lattwein K. R. Beekers I. Langeveld S. A. Leon-Grooters M. Strub J.-M. (2021). Vancomycin-decorated microbubbles as a theranostic agent for *Staphylococcus aureus* biofilms. Int. J. Pharm. 609, 121154. 10.1016/j.ijpharm.2021.121154 34624449

[B124] Kousa O. Walters R. W. Saleh M. Awad D. Qasim A. Guddeti R. R. (2020). Early vs late cardiac surgery in patients with native valve endocarditis-United States Nationwide Inpatient database. J. Card. Surg. 35, 2611–2617. 10.1111/jocs.14854 32720363

[B125] Kupferwasser L. I. Yeaman M. R. Shapiro S. M. Nast C. C. Sullam P. M. Filler S. G. (1999). Acetylsalicylic acid reduces vegetation bacterial density, hematogenous bacterial dissemination, and frequency of embolic events in experimental *Staphylococcus aureus* endocarditis through antiplatelet and antibacterial effects. Circulation 99, 2791–2797. 10.1161/01.cir.99.21.2791 10351974

[B126] Lancellotti P. Musumeci L. Jacques N. Servais L. Goffin E. Pirotte B. (2019). Antibacterial activity of ticagrelor in Conventional antiplatelet Dosages against antibiotic-resistant gram-positive bacteria. JAMA Cardiol. 4, 596–599. 10.1001/jamacardio.2019.1189 31066863 PMC6506905

[B127] Lanz J. Reardon M. J. Pilgrim T. Stortecky S. Deeb G. M. Chetcuti S. (2021). Incidence and outcomes of infective endocarditis after transcatheter or surgical aortic valve replacement. J. Am. Heart Assoc. 10, e020368. 10.1161/JAHA.120.020368 34581194 PMC8649131

[B128] Lattwein K. R. Beekers I. Kouijzer J. J. P. Leon-Grooters M. Langeveld S. A. G. van Rooij T. (2022). Dispersing and Sonoporating biofilm-associated bacteria with sonobactericide. Pharmaceutics 14. 10.3390/pharmaceutics14061164 PMC9227517 35745739

[B129] Lattwein K. R. Shekhar H. Kouijzer J. J. van Wamel W. J. Holland C. K. Kooiman K. (2020). Sonobactericide: An emerging treatment strategy for bacterial infections. Ultrasound Med. Biol. 46, 193–215. 10.1016/j.ultrasmedbio.2019.09.011 31699550 PMC9278652

[B130] Lattwein K. R. Shekhar H. van Wamel W. J. B. Gonzalez T. Herr A. B. Holland C. K. (2018). An in vitro proof-of-principle study of sonobactericide. Sci. Rep. 8, 3411. 10.1038/s41598-018-21648-8 29467474 PMC5821825

[B131] Lauten A. Martinović M. Kursawe L. Kikhney J. Affeld K. Kertzscher U. (2021). Bacterial biofilms in infective endocarditis: An in vitro model to investigate emerging technologies of antimicrobial cardiovascular device coatings. Clin. Res. Cardiol. 110, 323–331. 10.1007/s00392-020-01669-y 32444905 PMC7907033

[B132] Le Bot A. Lecomte R. Gazeau P. Benezit F. Arvieux C. Ansart S. (2021). Is Rifampin Use Associated With Better Outcome in Staphylococcal Prosthetic Valve Endocarditis? A Multicenter Retrospective Study, 72. Arlington: Clinical Infectious Diseases. 10.1093/cid/ciaa1040 32706879

[B133] Lebeaux D. Chauhan A. Rendueles O. Beloin C. (2013). From in vitro to in vivo models of bacterial biofilm-related infections. Pathogens 2, 288–356. 10.3390/pathogens2020288 25437038 PMC4235718

[B134] Leeten K. Jacques N. Lancellotti P. Oury C. (2021). Aspirin or ticagrelor in *Staphylococcus aureus* infective endocarditis: Where do We Stand? Front. Cell. Dev. Biol. 9, 716302. 10.3389/fcell.2021.716302 34692677 PMC8529053

[B135] Lehner A. Haas N. A. Dietl M. Jakob A. Schulze-Neick I. Dalla Pozza R. (2019). The risk of infective endocarditis following interventional pulmonary valve implantation: A meta-analysis. J. Cardiol. 74, 197–205. 10.1016/j.jjcc.2019.04.007 31113702

[B136] Lerche C. J. Christophersen L. J. Kolpen M. Nielsen P. R. Trøstrup H. Thomsen K. (2017). Hyperbaric oxygen therapy augments tobramycin efficacy in experimental *Staphylococcus aureus* endocarditis. Int. J. Antimicrob. Agents 50, 406–412. 10.1016/j.ijantimicag.2017.04.025 28669832

[B137] Lerche C. J. Schwartz F. Theut M. Fosbøl E. L. Iversen K. Bundgaard H. (2021). Anti-biofilm approach in infective endocarditis Exposes new treatment strategies for improved outcome. Front. Cell. Dev. Biol. 9, 643335. 10.3389/fcell.2021.643335 34222225 PMC8249808

[B138] Li J. Zhou L. Gong X. Wang Y. Yao D. Li H. (2022). Abiotrophia defectiva as a rare cause of mitral valve infective endocarditis with Mesenteric arterial Branch pseudoaneurysm, Splenic infarction, and renal infarction: A case report. Front. Med. 9, 780828. 10.3389/fmed.2022.780828 PMC8962948 35360716

[B139] Liesenborghs L. Meyers S. Lox M. Criel M. Claes J. Peetermans M. (2019). *Staphylococcus aureus* endocarditis: Distinct mechanisms of bacterial adhesion to damaged and inflamed heart valves. Eur. Heart J. 40, 3248–3259. 10.1093/eurheartj/ehz175 30945735 PMC7963134

[B140] Liesenborghs L. Meyers S. Vanassche T. Verhamme P. (2020). Coagulation: At the heart of infective endocarditis. J. Thromb. Haemost. 18, 995–1008. 10.1111/jth.14736 31925863

[B141] Liesman R. M. Pritt B. S. Maleszewski J. J. Patel R. (2017). Laboratory diagnosis of infective endocarditis. J. Clin. Microbiol. 55, 2599–2608. 10.1128/JCM.00635-17 28659319 PMC5648697

[B142] Lilje B. Rasmussen R. V. Dahl A. Stegger M. Skov R. L. Fowler V. G. (2017). Whole-genome sequencing of bloodstream *Staphylococcus aureus* isolates does not distinguish bacteraemia from endocarditis. Microb. Genom 3. 10.1099/mgen.0.000138 PMC5729915 29208121

[B143] Litzler P. Y. Benard L. Barbier-Frebourg N. Vilain S. Jouenne T. Beucher E. (2007). biofilm formation on pyrolytic carbon heart valves: Influence of surface free energy, roughness, and bacterial species. J. Thorac. Cardiovasc. Surg. 134, 1025–1032. 10.1016/j.jtcvs.2007.06.013 17903524

[B144] MacKintosh E. E. Patel J. D. Marchant R. E. Anderson J. M. (2006). Effects of biomaterial surface chemistry on the adhesion and biofilm formation of Staphylococcus epidermidis in vitro. J. Biomed. Mat. Res. 78A, 836–842. 10.1002/jbm.a.30905 16817192

[B145] Maharaj B. Parrish A. (2012). Prevention of infective endocarditis in developing countries. Cvja 23, 303–305. 10.5830/cvja-2012-004 22836149 PMC3721954

[B146] Małecka B. Ząbek A. Dębski M. Szot W. Holcman K. Boczar K. (2018). The usefulness of SPECT-CT with radioisotope-labeled leukocytes in diagnosing lead-dependent infective endocarditis. Adv. Clin. Exp. Med. 28. 10.17219/acem/92315 30411545

[B147] Marchetta S. Withofs N. Erba P. A. Habib G. Hustinx R. Lancellotti P. (2017). Radionuclide imaging of infective endocarditis: State of Art and future Perspective. Curr. Cardiovasc. Imaging Rep. 10. 10.1007/s12410-017-9425-1

[B148] Marrie T. J. Costerton J. W. (1984). Morphology of bacterial attachment to cardiac pacemaker leads and power packs. J. Clin. Microbiol. 19, 911–914. 10.1128/jcm.19.6.911-914.1984 6470101 PMC271211

[B149] Martin D. R. Witten J. C. Tan C. D. Rodriguez E. R. Blackstone E. H. Pettersson G. B. (2020). Proteomics identifies a convergent innate response to infective endocarditis and extensive proteolysis in vegetation components. JCI Insight 5. 10.1172/jci.insight.135317 PMC7453909 32544089

[B150] Martini A. M. Moricz B. S. Ripperger A. K. Tran P. M. Sharp M. E. Forsythe A. N. (2020). Association of novel Streptococcus sanguinis virulence factors with pathogenesis in a native valve infective endocarditis model. Front. Microbiol. 11, 10. 10.3389/fmicb.2020.00010 32082276 PMC7005726

[B151] Mateos Gaitán R. Boix-Palop L. Muñoz García P. Mestres C. A. Marín Arriaza M. Pedraz Prieto Á. (2020). Infective endocarditis in patients with cardiac implantable electronic devices: a nationwide study, 22. EP Europace. 10.1093/europace/euaa076 32390046

[B152] Memmott M. J. James J. Armstrong I. S. Tout D. Ahmed F. (2016). The performance of quantitation methods in the evaluation of cardiac implantable electronic device (cied) infection: A technical review. J. Nucl. Cardiol. 23, 1457–1466. 10.1007/s12350-015-0106-7 26403147

[B153] Michałowska I. Stokłosa P. Miłkowska M. Zakrzewski D. Nieznańska M. Kwiatek P. (2021). The role of cardiac computed tomography in the diagnosis of prosthetic valve endocarditis - a comparison with transthoracic and transesophageal echocardiography and intra-operative findings. Eur. J. Radiol. 138, 109637. 10.1016/j.ejrad.2021.109637 33740628

[B154] Millar B. Moore J. Mallon P. Xu J. Crowe M. Mcclurg R. (2001). Molecular diagnosis of infective endocarditis--a new Duke's criterion. Scand. J. Infect. Dis. 33, 673–680. 10.1080/00365540110026764 11669225

[B155] Mistiaen W. P. (2018). What are the main predictors of in-hospital mortality in patients with infective endocarditis: A review. Scand. Cardiovasc J. 52, 58–68. 10.1080/14017431.2018.1433318 29382232

[B156] Miyagi Y. Kawase Y. Kunugi S. Oomori H. Sasaki T. Sakamoto S. I. (2020). Histological properties of oscillating intracardiac masses associated with cardiac implantable electric devices. J. Arrhythmia 36, 478–484. 10.1002/joa3.12346 PMC7279976 32528575

[B157] Munita J. M. Arias C. A. Murray B. E. (2012). Enterococcal endocarditis: Can We Win the war? Curr. Infect. Dis. Rep. 14, 339–349. 10.1007/s11908-012-0270-8 22661339 PMC4433165

[B158] Nair S. Desai S. Poonacha N. Vipra A. Sharma U. (2016). Antibiofilm activity and synergistic inhibition of *Staphylococcus aureus* biofilms by bactericidal protein P128 in combination with antibiotics. Antimicrob. Agents Chemother. 60, 7280–7289. 10.1128/aac.01118-16 27671070 PMC5119008

[B159] Narducci M. L. Pelargonio G. Russo E. Marinaccio L. Di Monaco A. Perna F. (2013). Usefulness of intracardiac echocardiography for the diagnosis of cardiovascular implantable electronic device-related endocarditis. J. Am. Coll. Cardiol. 61, 1398–1405. 10.1016/j.jacc.2012.12.041 23500279

[B160] Nasso G. Santarpino G. Moscarelli M. Condello I. Dell’Aquila A. M. Peivandi A. D. (2021). Surgical treatment of valve endocarditis in high-risk patients and predictors of long-term outcomes. Sci. Rep. 11, 24223. 10.1038/s41598-021-03602-3 34930958 PMC8688441

[B161] Nesterovics N. Nesterovics G. Stradins P. Kalejs M. Ansabergs J. Blumbergs M. (2019). Lead-Related Infective Endocarditis in Latvia: A Single Centre Experience, 55. Medicina. 10.3390/medicina55090566 PMC6780120 31484433

[B162] Nguyen A. K. Palafox B. A. Starr J. P. Gates R. N. Berdjis F. (2016). Endocarditis and incomplete Endothelialization 12 Years after Amplatzer septal occluder deployment. Tex Heart Inst. J. 43, 227–231. 10.14503/thij-14-4949 27303238 PMC4894701

[B163] Nishiguchi S. Nishino K. Kitagawa I. Tokuda Y. (2020)., 99. Medicine, e21418. 10.1097/MD.0000000000021418 Factors associated with delayed diagnosis of infective endocarditis: A retrospective cohort study in a teaching hospital in Japan Med. Baltim. PMC7386977 32791760

[B164] Noriaki M. Teemu L. Fausto B. Peter R. Maina P. J. Jussi J. (2019). Prosthetic valve endocarditis after transcatheter or surgical aortic valve replacement with a bioprosthesis: Results from the FinnValve registry. EuroIntervention 15, e500–e507. 31113766 10.4244/EIJ-D-19-00247

[B165] Oberbach A. Schlichting N. Feder S. Lehmann S. Kullnick Y. Buschmann T. (2017). New insights into valve-related intramural and intracellular bacterial diversity in infective endocarditis. PLoS One 12, e0175569. 10.1371/journal.pone.0175569 28410379 PMC5391965

[B166] Oh T. H. T. Wang T. K. M. Pemberton J. A. Raudkivi P. J. (2016). Early or late surgery for endocarditis with neurological complications. Asian Cardiovasc Thorac. Ann. 24, 435–440. 10.1177/0218492316646903 27122616

[B167] Ojha N. Dhamoon A. S. (2022). Fungal endocarditis. *StatPearls.* Treasure. Island (FL. 30422581

[B168] Okita Y. Minakata K. Yasuno S. Uozumi R. Sato T. Ueshima K. (2016). Optimal timing of surgery for active infective endocarditis with cerebral complications: A Japanese multicentre study. Eur. J. Cardiothorac. Surg. 50, 374–382. 10.1093/ejcts/ezw035 26968761

[B169] Ortiz-Bautista C. López J. García-Granja P. E. Vilacosta I. Sevilla T. Sarriá C. (2017). Endocarditis infecciosa derecha en portadores de dispositivos cardiacos: perfil clínico y pronóstico, 149. Barcelona: Medicina Clínica. 10.1016/j.medcli.2017.03.055 28648588

[B170] Özcan C. Raunsø J. Lamberts M. Køber L. Lindhardt T. B. Bruun N. E. (2017). Infective endocarditis and risk of death after cardiac implantable electronic device implantation: A nationwide cohort study. EP Eur. 19. 10.1093/europace/euw404 28073883

[B171] Pabst R. (2020). The pig as a model for immunology research. Cell. Tissue Res. 380, 287–304. 10.1007/s00441-020-03206-9 32356014 PMC7223737

[B172] Panizzi P. Krohn-Grimberghe M. Keliher E. Ye Y. X. Grune J. Frodermann V. (2020). Multimodal imaging of bacterial-host interface in mice and piglets with *Staphylococcus aureus* endocarditis. Sci. Transl. Med. 12. 10.1126/scitranslmed.aay2104 PMC7818516 33148623

[B173] Pant S. Deshmukh N. J. Mehta A. Golwala H. Patel N. Badheka A. (2015). Reply: Trends in infective endocarditis: Incidence, microbiology, and valve replacement in the United States from 2000 to 2011: The Devil is in the Details. J. Am. Coll. Cardiol. 66, 1202–1203. 10.1016/j.jacc.2015.06.1330 26338003

[B174] Park B. S. Lee W. Y. Ra Y. J. Lee H. K. Gu B. M. Yang J. T. (2020). Surgical outcomes for native valve endocarditis. Korean J. Thorac. Cardiovasc Surg. 53, 1–7. 10.5090/kjtcs.2020.53.1.1 32090051 PMC7006608

[B175] Pasqualotto A. C. Denning D. W. (2006). Post-operative aspergillosis. Clin. Microbiol. Infect. 12, 1060–1076. 10.1111/j.1469-0691.2006.01512.x 17002605

[B176] Peetermans M. Liesenborghs L. Peerlinck K. Wijngaerden E. V. Gheysens O. Goffin K. E. (2018). Targeting Coagulase activity in *Staphylococcus aureus* bacteraemia: A randomized controlled single-centre trial of Staphylothrombin inhibition. Thromb. Haemost. 118, 818–829. 10.1055/s-0038-1639586 29614521

[B177] Pericàs J. M. Hernández-Meneses M. Muñoz P. Martínez-Sellés M. Álvarez-Uria A. de Alarcón A. (2021). Characteristics and outcome of acute heart failure in infective endocarditis: Focus on Cardiogenic shock. Clin. Infect. Dis. 73, 765–774. 10.1093/cid/ciab098 33560404

[B178] Petrovic Fabijan A. Lin R. C. Y. Ho J. Maddocks S. Ben Zakour N. L. Iredell J. R. (2020). Safety of bacteriophage therapy in severe *Staphylococcus aureus* infection. Nat. Microbiol. 5, 465–472. 10.1038/s41564-019-0634-z 32066959

[B179] Pettersson G. B. Hussain S. T. (2019). Current AATS guidelines on surgical treatment of infective endocarditis. Ann. Cardiothorac. Surg. 8, 630–644. 10.21037/acs.2019.10.05 31832353 PMC6892713

[B180] Pettersson G. B. Hussain S. T. Shrestha N. K. Gordon S. Fraser T. G. Ibrahim K. S. (2014). Infective endocarditis: An atlas of disease progression for describing, staging, coding, and understanding the pathology. J. Thorac. Cardiovasc. Surg. 147, 1142–1149. 10.1016/j.jtcvs.2013.11.031 24507402

[B181] Płońska-Gościniak E. Olędzki S. Kukulski T. Kosmala W. Młynarski R. Oko-Sarnowska Z. (2019). Pol-CDRIE registry – 1-year observational data on patients hospitalized due to cardiac device-related infective endocarditis in Polish referential cardiology centres, 77. Warsaw: Kardiologia Polska. 10.33963/KP.14811 31066721

[B182] Plumet L. Ahmad-Mansour N. Dunyach-Remy C. Kissa K. Sotto A. Lavigne J. P. (2022). Bacteriophage therapy for Staphylococcus aureus infections: A review of animal models, treatments, and clinical trials. Front. Cell. Infect. Microbiol. 12, 907314. 10.3389/fcimb.2022.907314 35782148 PMC9247187

[B183] Prudent E. Lepidi H. Angelakis E. Raoult D. (2018). Fluorescence *in situ* hybridization (FISH) and peptide nucleic acid probe-based FISH for diagnosis of Q fever endocarditis and vascular infections. J. Clin. Microbiol. 56, 005422–e618. 10.1128/JCM.00542-18 PMC6113452 29899006

[B184] Pyo W. K. Kim H. J. Kim J. B. Jung S.-H. Choo S. J. Chung C. H. (2021). Comparative surgical outcomes of prosthetic and native valve endocarditis. Korean Circ. J. 51, 504–514. 10.4070/kcj.2020.0448 34085423 PMC8176072

[B185] Qi X. Zhao Y. Zhang J. Han D. Chen C. Huang Y. (2016). Increased effects of Extracorporeal shock waves combined with gentamicin against *Staphylococcus aureus* biofilms in vitro and in vivo. Ultrasound Med. Biol. 42, 2245–2252. 10.1016/j.ultrasmedbio.2016.04.018 27260244

[B186] Rajani R. Klein J. L. (2020). Infective endocarditis: A contemporary update. Clin. Med. (Lond) 20, 31–35. 10.7861/clinmed.cme.20.1.1 31941729 PMC6964163

[B187] Ramos-Martínez A. Muñoz Serrano A. de Alarcón González A. Muñoz P. Fernández-Cruz A. Valerio M. (2018). Gentamicin may have no effect on mortality of staphylococcal prosthetic valve endocarditis. J. Infect. Chemother. 24. 10.1016/j.jiac.2018.03.003 29628387

[B188] Rezar R. Lichtenauer M. Haar M. Hödl G. Kern J. M. Zhou Z. (2021). Infective endocarditis - a review of current therapy and future challenges. Hell. J. Cardiol. 62, 190–200. 10.1016/j.hjc.2020.10.007 33176209

[B189] Roder C. Gunjaca V. Otome O. Gwini S. M. Athan E. (2020). Cost and outcomes of implantable cardiac electronic device infections in victoria, Australia. Heart Lung Circ. 29, e140. 10.1016/j.hlc.2019.10.010 31839364

[B190] Rosenbaum S. J. Lind T. Antoch G. Bockisch A. (2006). False-positive FDG PET Uptake−the role of PET/CT. Eur. Radiol. 16, 1054–1065. 10.1007/s00330-005-0088-y 16365730

[B191] Said S. M. Abdelsattar Z. M. Schaff H. V. Greason K. L. Daly R. C. Pochettino A. (2018). Outcomes of surgery for infective endocarditis: A single-centre experience of 801 patients. Eur. J. Cardiothorac. Surg. 53, 435–439. 10.1093/ejcts/ezx341 29029030

[B192] Salaun E. Deharo J. C. Casalta J. P. Franceschi F. Hubert S. Renard S. (2017). An oscillating mass attached to a pacemaker lead. JACC Clin. Electrophysiol. 3, 915–916. 10.1016/j.jacep.2016.12.028 29759791

[B193] Salgado-Pabón W. Schlievert P. M. (2014). Models matter: The search for an effective *Staphylococcus aureus* vaccine. Nat. Rev. Microbiol. 12, 585–591. 10.1038/nrmicro3308 24998740

[B194] Salomäki S. P. Saraste A. Kemppainen J. Bax J. J. Knuuti J. Nuutila P. (2017). 18F-FDG positron emission tomography/computed tomography in infective endocarditis. J. Nucl. Cardiol. 24, 195–206. 10.1007/s12350-015-0325-y 26662063

[B195] Samura T. Yoshioka D. Toda K. Sakaniwa R. Yokoyama J. Suzuki K. (2019). Emergency valve surgery improves clinical results in patients with infective endocarditis complicated with acute cerebral infarction: Analysis using propensity score matching†. Eur. J. Cardiothorac. Surg. 56, 942–949. 10.1093/ejcts/ezz100 31502643

[B196] San S. Ravis E. Tessonier L. Philip M. Cammilleri S. Lavagna F. (2019). Prognostic value of 18F-fluorodeoxyglucose positron emission tomography/computed tomography in infective endocarditis. J. Am. Coll. Cardiol. 74, 1031–1040. 10.1016/j.jacc.2019.06.050 31439211

[B197] Sánchez-Enrique C. Olmos C. Jiménez-Ballvé A. Fernández-Pérez C. Ferrera C. Pérez-Castejón M. J. (2018). Usefulness of 18F fluorodeoxyglucose positron emission tomography/computed tomography in infective endocarditis in Daily Practice. JACC Cardiovasc. Imaging 11. 10.1016/j.jcmg.2018.06.011 30121263

[B198] Sande M. A. (1999). Handbook of animal models of infection: Experimental models in antimicrobial chemotherapy. Academic Press.

[B199] Schwartz F. A. Christophersen L. Laulund A. S. Lundquist R. Lerche C. Rude Nielsen P. (2021). Novel human in vitro vegetation simulation model for infective endocarditis. APMIS 129, 653–662. 10.1111/apm.13182 34580927

[B200] Schwarz C. Buchholz R. Jawad M. Hoesker V. Terwesten-Solé C. Karst U. (2022). Fingerprints of element concentrations in infective endocarditis obtained by mass spectrometric imaging and t-Distributed Stochastic neighbor embedding. ACS Infect. Dis. 8, 360–372. 10.1021/acsinfecdis.1c00485 35045258

[B201] Schwarz C. Hoerr V. Töre Y. Hösker V. Hansen U. Van de Vyver H. (2020). Isolating crucial Steps in induction of infective endocarditis with preclinical modeling of host pathogen interaction. Front. Microbiol. 11, 1325. 10.3389/fmicb.2020.01325 32625192 PMC7314968

[B202] Schwarz C. Töre Y. Hoesker V. Ameling S. Grün K. Völker U. (2021). Host-pathogen interactions of clinical *S. aureus* isolates to induce infective endocarditis. Virulence 12, 2073–2087. 10.1080/21505594.2021.1960107 34490828 PMC8425731

[B203] Sedgwick J. F. Scalia G. M. (2016). Advanced echocardiography for the diagnosis and management of infective endocarditis. *Contemporary Challenges in endocarditis* . London: InTech.

[B204] Seelig M. S. Speth C. P. Kozinn P. J. Taschdjian C. L. Toni E. F. Goldberg P. (1974). Patterns of Candida endocarditis following cardiac surgery: Importance of early diagnosis and therapy (an analysis of 91 cases). Prog. Cardiovasc. Dis. 17, 125–160. 10.1016/0033-0620(74)90027-9 4853560

[B205] Sekar P. Johnson J. R. Thurn J. R. Drekonja D. M. Morrison V. A. Chandrashekhar Y. (2017). Comparative sensitivity of transthoracic and transesophageal echocardiography in diagnosis of infective endocarditis among veterans with *Staphylococcus aureus* bacteremia. Open Forum Infect. Dis. 4, ofx035. 10.1093/ofid/ofx035 28470017 PMC5407216

[B206] Shah A. S. V. McAllister D. A. Gallacher P. Astengo F. Rodríguez Pérez J. A. Hall J. (2020a). Incidence, microbiology, and outcomes in patients hospitalized with infective endocarditis. Circulation 141, 2067–2077. 10.1161/CIRCULATIONAHA.119.044913 32410460 PMC7306256

[B207] Shah S. U. Xiong Y. Q. Abdelhady W. Iwaz J. Pak Y. Schuch R. (2020b). Effect of the lysin exebacase on cardiac vegetation progression in a rabbit model of methicillin-resistant *Staphylococcus aureus* endocarditis as determined by echocardiography. Antimicrob. Agents Chemother. 64. 10.1128/AAC.00482-20 PMC7317998 32340988

[B208] Shokohi T. Nouraei S. M. Afsarian M. H. Najafi N. Mehdipour S. (2014). Fungal prosthetic valve endocarditis by Candida parapsilosis: A case report. Jundishapur J. Microbiol. 7, e9428. 10.5812/jjm.9428 25147692 PMC4138648

[B209] Shrestha N. Shakya S. Hussain S. Pettersson G. Griffin B. Gordon S. (2017). Sensitivity and Specificity of Duke Criteria for Diagnosis of Definite Infective Endocarditis: A Cohort Study, 4. Arlington: Open Forum Infectious Diseases. 10.1093/ofid/ofx163.1431

[B210] Sifaoui I. Oliver L. Tacher V. Fiore A. Lepeule R. Moussafeur A. (2020). Diagnostic performance of transesophageal echocardiography and cardiac computed tomography in infective endocarditis. J. Am. Soc. Echocardiogr. 33, 1442–1453. 10.1016/j.echo.2020.07.017 32981789

[B211] Singhal N. Kumar M. Kanaujia P. K. Virdi J. S. (2015). MALDI-TOF mass spectrometry: An emerging technology for microbial identification and diagnosis. Front. Microbiol. 6, 791. 10.3389/fmicb.2015.00791 26300860 PMC4525378

[B212] Sivak J. A. Vora A. N. Navar A. M. Schulte P. J. Crowley A. L. Kisslo J. (2016). An approach to improve the negative predictive value and clinical utility of transthoracic echocardiography in suspected native valve infective endocarditis. J. Am. Soc. Echocardiogr. 29, 315–322. 10.1016/j.echo.2015.12.009 26850679 PMC6052444

[B213] Sławiński G. Lewicka E. Kempa M. Budrejko S. Raczak G. (2019). Infections of cardiac implantable electronic devices: Epidemiology, classification, treatment, and prognosis. Adv. Clin. Exp. Med. 28, 263–270. 10.17219/acem/80665 30048058

[B214] Snipsøyr M. G. Wiggers H. Ludvigsen M. Stensballe A. Vorum H. Poulsen S. H. (2020). Towards identification of novel putative biomarkers for infective endocarditis by serum proteomic analysis. Int. J. Infect. Dis. 96, 73–81. 10.1016/j.ijid.2020.02.026 32087365

[B215] Sordelli C. Fele N. Mocerino R. Weisz S. Ascione L. Caso P. (2019). Infective endocarditis: Echocardiographic imaging and new imaging modalities. J. Cardiovasc Echogr 29, 149–155. 10.4103/jcecho.jcecho_53_19 32089994 PMC7011492

[B216] Srinivasan M. Sedmak D. Jewell S. (2002). Effect of fixatives and tissue processing on the content and integrity of nucleic acids. Am. J. Pathology 161, 1961–1971. 10.1016/s0002-9440(10)64472-0 PMC1850907 12466110

[B217] Summers M. R. Leon M. B. Smith C. R. Kodali S. K. Thourani V. H. Herrmann H. C. (2019). Prosthetic valve endocarditis after TAVR and SAVR. Circulation 140, 1984–1994. 10.1161/circulationaha.119.041399 31690104

[B218] Suzuki K. Yoshioka D. Toda K. Yokoyama J.-Y. Samura T. Miyagawa S. (2019). Results of surgical management of infective endocarditis associated with *Staphylococcus aureus* . Eur. J. Cardiothorac. Surg. 56, 30–37. 10.1093/ejcts/ezy470 30689791

[B219] Tak T. Shukla S. K. (2004). Molecular diagnosis of infective endocarditis: A helpful addition to the Duke criteria. Clin. Med. Res. 2, 206–208. 10.3121/cmr.2.4.206 15931359 PMC1069095

[B220] Talha K. M. DeSimone D. C. Sohail M. R. Baddour L. M. (2020). Pathogen influence on epidemiology, diagnostic evaluation and management of infective endocarditis. Heart 106, 1878–1882. 10.1136/heartjnl-2020-317034 32847941

[B221] Teoh T. K. Hannan M. M. (2018). Ventricular assist device-associated infection. Infect. Dis. Clin. N. Am. 32, 827–841. 10.1016/j.idc.2018.07.001 30241710

[B222] Thornhill M. H. Dayer M. J. Nicholl J. Prendergast B. D. Lockhart P. B. Baddour L. M. (2020). An alarming rise in incidence of infective endocarditis in England since 2009: Why? Lancet 395, 1325–1327. 10.1016/S0140-6736(20)30530-4 32334690

[B223] Thornhill M. H. Gibson T. B. Cutler E. Dayer M. J. Chu V. H. Lockhart P. B. (2018). Antibiotic prophylaxis and incidence of endocarditis before and after the 2007 AHA recommendations. J. Am. Coll. Cardiol. 72, 2443–2454. 10.1016/j.jacc.2018.08.2178 30409564

[B224] Thornhill M. H. Gibson T. B. Yoon F. Dayer M. J. Prendergast B. D. Lockhart P. B. (2022). Antibiotic prophylaxis Against infective endocarditis before invasive dental procedures. J. Am. Coll. Cardiol. 10.1016/j.jacc.2022.06.030

[B225] Thwaites G. E. Scarborough M. Szubert A. Nsutebu E. Tilley R. Greig J. (2018). Adjunctive rifampicin for *Staphylococcus aureus* bacteraemia (ARREST): A multicentre, randomised, double-blind, placebo-controlled trial. Lancet 391, 668–678. 10.1016/S0140-6736(17)32456-X 29249276 PMC5820409

[B226] To R. K. Ramchandar N. Gupta A. Pong A. Cannavino C. Foley J. (2021). Use of plasma metagenomic next-generation sequencing for pathogen identification in pediatric endocarditis. Pediatr. Infect. Dis. J. 40, 486–488. 10.1097/inf.0000000000003038 33410648

[B227] Tong S. Y. Davis J. S. Eichenberger E. Holland T. L. Fowler V. G., JR. (2015). *Staphylococcus aureus* infections: Epidemiology, pathophysiology, clinical manifestations, and management. Clin. Microbiol. Rev. 28, 603–661. 10.1128/cmr.00134-14 26016486 PMC4451395

[B228] Toyoda N. Itagaki S. Egorova N. N. Tannous H. Anyanwu A. C. El-Eshmawi A. (2017). Real-world outcomes of surgery for native mitral valve endocarditis. J. Thorac. Cardiovasc Surg. 154, 1906–e9. 10.1016/j.jtcvs.2017.07.077 28942975

[B229] Trübe P. Hertlein T. Mrochen D. M. Schulz D. Jorde I. Krause B. (2019). Bringing together what belongs together: Optimizing murine infection models by using mouse-adapted *Staphylococcus aureus* strains. Int. J. Med. Microbiol. 309, 26–38. 10.1016/j.ijmm.2018.10.007 30391222

[B230] Vakilzadeh J. Rowlands D. T., JR. Sherwood B. F. LeMay J. C. (1970). Experimental bacterial endocarditis in the opossum (*Didelphis virginiana*). J. Infect. Dis. 122, 89–92. 10.1093/infdis/122.1-2.89 5433712

[B231] van den Brink F. S. Swaans M. J. Hoogendijk M. G. Alipour A. Kelder J. C. Jaarsma W. (2017). Increased incidence of infective endocarditis after the 2009 European Society of cardiology guideline update: A nationwide study in The Netherlands. Eur. Heart J. Qual. Care Clin. Outcomes 3, 141–147. 10.1093/ehjqcco/qcw039 28927175

[B232] van der vaart T. W. Deckers J. W. Natour E. H. Verkaik N. J. van der Meer J. T. M. (2019). SWAB guidelines for the antimicrobial treatment of infective endocarditis. [Online]. Available:[Accessed] https://swab.nl/nl/exec/file/download/152.

[B233] Velangi P. S. Kalra R. Markowitz J. Nijjar P. S. (2020). Utility of CT in the diagnosis of prosthetic valve abnormalities. J. Card. Surg. 35, 3025–3033. 10.1111/jocs.14966 32827165

[B234] Veloso T. R. Amiguet M. Rousson V. Giddey M. Vouillamoz J. Moreillon P. (2011). Induction of experimental endocarditis by continuous low-grade bacteremia mimicking spontaneous bacteremia in humans. Infect. Immun. 79, 2006–2011. 10.1128/iai.01208-10 21321073 PMC3088130

[B235] Veloso T. R. Que Y. A. Chaouch A. Giddey M. Vouillamoz J. Rousson V. (2015). Prophylaxis of experimental endocarditis with antiplatelet and antithrombin agents: A role for long-term prevention of infective endocarditis in humans? J. Infect. Dis. 211, 72–79. 10.1093/infdis/jiu426 25086177

[B236] Vincent L. L. Otto C. M. (2018). Infective endocarditis: Update on epidemiology, outcomes, and management. Curr. Cardiol. Rep. 20, 86. 10.1007/s11886-018-1043-2 30117004

[B237] Vollmer T. Piper C. Horstkotte D. Korfer R. Kleesiek K. Dreier J. (2010). 23S rDNA real-time polymerase chain reaction of heart valves: A decisive tool in the diagnosis of infective endocarditis. Eur. Heart J. 31, 1105–1113. 10.1093/eurheartj/ehp600 20093256

[B238] wahadat A. R. Tanis W. Galema T. W. Swart L. E. van Leeuwen W. J. Verkaik N. J. (2022). The impact of the multidisciplinary Endocarditis Team on the management of infective endocarditis. Neth Heart J. 10.1007/s12471-022-01707-6 PMC9807728 35781784

[B239] Wang A. Chu V. H. Athan E. Delahaye F. Freiberger T. Lamas C. (2019). Association between the timing of surgery for complicated, left-sided infective endocarditis and survival. Am. Heart J. 210, 108–116. 10.1016/j.ahj.2019.01.004 30802708

[B240] Wang A. Gaca J. G. Chu V. H. (2018a). Management considerations in infective endocarditis: A review. JAMA 320, 72–83. 10.1001/jama.2018.7596 29971402

[B241] Wang F. Zhou H. Olademehin O. P. Kim S. J. Tao P. (2018b). Insights into key interactions between vancomycin and bacterial cell wall structures. ACS Omega 3, 37–45. 10.1021/acsomega.7b01483 29399648 PMC5793038

[B242] Wang T. K. M. Sánchez-Nadales A. Igbinomwanhia E. Cremer P. Griffin B. Xu B. (2020). Diagnosis of infective endocarditis by Subtype using 18F-fluorodeoxyglucose positron emission tomography/computed tomography: A contemporary meta-analysis. Circ. Cardiovasc Imaging 13, e010600. 10.1161/CIRCIMAGING.120.010600 32507019

[B243] Wardak M. Gowrishankar G. Zhao X. Liu Y. Chang E. Namavari M. (2020). Molecular imaging of infective endocarditis with 6′′-[ 18 F]Fluoromaltotriose positron emission tomography-computed tomography. Circulation 141, 1729–1731. 10.1161/circulationaha.119.043924 32453662 PMC9183944

[B244] Watson A. Sauve K. Cassino C. Schuch R. (2020). Exebacase Demonstrates in vitro Synergy with a broad range of antibiotics against both methicillin-resistant and methicillin-Susceptible *Staphylococcus aureus* . Antimicrob. Agents Chemother. 64, 018855–e1919. 10.1128/AAC.01885-19 PMC6985718 31712212

[B245] Weber C. Petrov G. Luehr M. Aubin H. Tugtekin S.-M. Borger M. A. (2021). Surgical results for prosthetic versus native valve endocarditis: A multicenter analysis. J. Thorac. Cardiovasc Surg. 161, 609–e10. 10.1016/j.jtcvs.2019.09.186 31780064

[B246] Werdan K. Dietz S. Löffler B. Niemann S. Bushnaq H. Silber R. E. (2014). Mechanisms of infective endocarditis: Pathogen-host interaction and risk states. Nat. Rev. Cardiol. 11, 35–50. 10.1038/nrcardio.2013.174 24247105

[B247] Williams M. L. Doyle M. P. McNamara N. Tardo D. Mathew M. Robinson B. (2021). Epidemiology of infective endocarditis before versus after change of international guidelines: A systematic review. Ther. Adv. Cardiovasc Dis. 15, 17539447211002687. 10.1177/17539447211002687 33784909 PMC8020745

[B248] Wilson W. Taubert K. A. Gewitz M. Lockhart P. B. Baddour L. M. Levison M. (2007). Prevention of infective endocarditis: Guidelines from the American heart association: A guideline from the American heart association rheumatic fever, endocarditis, and kawasaki disease Committee, Council on cardiovascular disease in the young, and the Council on clinical cardiology, Council on cardiovascular surgery and Anesthesia, and the quality of care and outcomes research interdisciplinary working group. Circulation 116, 1736–1754. 10.1161/CIRCULATIONAHA.106.183095 17446442

[B249] Witten J. C. Hussain S. T. Shrestha N. K. Gordon S. M. Houghtaling P. L. Bakaeen F. G. (2019). Surgical treatment of right-sided infective endocarditis. J. Thorac. Cardiovasc Surg. 157, 1418–e14. 10.1016/j.jtcvs.2018.07.112 30503743

[B250] Wu Z. Chen Y. Xiao T. Niu T. Shi Q. Xiao Y. (2019). The clinical features and prognosis of infective endocarditis in the elderly from 2007 to 2016 in a tertiary hospital in China. BMC Infect. Dis. 19, 937. 10.1186/s12879-019-4546-6 31694555 PMC6836522

[B251] Xiong Y. Q. Willard J. Kadurugamuwa J. L. Yu J. Francis K. P. Bayer A. S. (2005). Real-time in vivo bioluminescent imaging for evaluating the efficacy of antibiotics in a rat *Staphylococcus aureus* endocarditis model. Antimicrob. Agents Chemother. 49, 380–387. 10.1128/aac.49.1.380-387.2005 15616318 PMC538900

[B252] Yang E. Frazee B. W. (2018). Infective endocarditis. Philadelphia: Emergency Medicine Clinics of North America, 36. 10.1016/j.emc.2018.06.002 30296997

[B253] Yang Y. C. Aung T. T. Khan S. Wase A. (2019). Utility of intracardiac echocardiography to diagnose infective endocarditis. J. Investig. Med. High. Impact Case Rep. 7, 2324709618822075. 10.1177/2324709618822075 PMC6350115 30791720

[B254] Yuan S. M. (2016). Fungal endocarditis. Braz J. Cardiovasc Surg. 31, 252–255. 10.5935/1678-9741.20160026 27737409 PMC5062704

[B255] Zeng X. Wu J. Li X. Xiong W. Tang L. Li X. (2022). Application of metagenomic next-generation sequencing in the etiological diagnosis of infective endocarditis during the Perioperative Period of cardiac surgery: A prospective cohort study. Front. Cardiovasc. Med. 9, 811492. 10.3389/fcvm.2022.811492 35369282 PMC8965566

